# Rosetta FunFolDes – A general framework for the computational design of functional proteins

**DOI:** 10.1371/journal.pcbi.1006623

**Published:** 2018-11-19

**Authors:** Jaume Bonet, Sarah Wehrle, Karen Schriever, Che Yang, Anne Billet, Fabian Sesterhenn, Andreas Scheck, Freyr Sverrisson, Barbora Veselkova, Sabrina Vollers, Roxanne Lourman, Mélanie Villard, Stéphane Rosset, Thomas Krey, Bruno E. Correia

**Affiliations:** 1 Institute of Bioengineering, École Polytechnique Fédérale de Lausanne, Lausanne, Switzerland; 2 Swiss Institute of Bioinformatics (SIB), Lausanne, Switzerland; 3 Institute of Virology, Hannover Medical School, Hannover, Germany; 4 German Center for Infection Research (DZIF), Hannover, Germany; Weizmann Institute of Science, ISRAEL

## Abstract

The robust computational design of functional proteins has the potential to deeply impact translational research and broaden our understanding of the determinants of protein function and stability. The low success rates of computational design protocols and the extensive *in vitro* optimization often required, highlight the challenge of designing proteins that perform essential biochemical functions, such as binding or catalysis. One of the most simplistic approaches for the design of function is to adopt functional motifs in naturally occurring proteins and transplant them to computationally designed proteins. The structural complexity of the functional motif largely determines how readily one can find host protein structures that are “designable”, meaning that are likely to present the functional motif in the desired conformation. One promising route to enhance the “designability” of protein structures is to allow backbone flexibility. Here, we present a computational approach that couples conformational folding with sequence design to embed functional motifs into heterologous proteins—Rosetta Functional Folding and Design (FunFolDes). We performed extensive computational benchmarks, where we observed that the enforcement of functional requirements resulted in designs distant from the global energetic minimum of the protein. An observation consistent with several experimental studies that have revealed function-stability tradeoffs. To test the design capabilities of FunFolDes we transplanted two viral epitopes into distant structural templates including one *de novo* “functionless” fold, which represent two typical challenges where the designability problem arises. The designed proteins were experimentally characterized showing high binding affinities to monoclonal antibodies, making them valuable candidates for vaccine design endeavors. Overall, we present an accessible strategy to repurpose old protein folds for new functions. This may lead to important improvements on the computational design of proteins, with structurally complex functional sites, that can perform elaborate biochemical functions related to binding and catalysis.

## Author summary

The ability to use computational tools to manipulate the structure and function of proteins has the potential to impact many facets of fundamental and translational science. Due to our limited understanding of the principles that govern protein function and structure, the computational design of functional proteins remains challenging. We developed a computational protocol (Rosetta FunFolDes) to facilitate the insertion of functional motifs into heterologous proteins. We performed extensive *in silico* benchmarks, and found that when the design of function is required the global energy minima may not be the optimal solution, in line with previously reported experimental studies. Further, we used FunFolDes to design two novel functional proteins, displaying two viral epitopes that can be of interest for vaccine development. The designed proteins were experimentally characterized, showing that functionalization was successfully achieved. These results highlight the capability of FunFolDes to address common challenges on the design of functional proteins. In particular, the reduced structural compatibility between functional sites and host scaffolds, effectively enabling the repurposing of old protein folds for new functions. Overall, FunFolDes provides new means to accomplish the challenging task of functionalizing computationally designed proteins.

## Introduction

Proteins are one of the main functional building blocks of the cell. The ability to create novel proteins outside of the natural realm has opened the path towards innovative achievements, such as new pathways [1], cellular functions [2], and therapeutic leads [3–5]. Computational protein design is the rational and structure-based approach to solve the inverse folding problem, i.e. the search for the best putative sequence capable of fitting and stabilizing a protein’s three-dimensional conformation [6]. As such, a great deal of effort has been placed into understanding the rules of protein folding and stability [7, 8] and its relation to the appropriate sequence space [9].

Computational protein design approaches focus on exploring two interconnected landscapes related to sampling of the conformational and sequence spaces. Fixed backbone approaches use static protein backbone conformations, which greatly constrain the sequence space explored by the computational algorithm [9]. Following the same principles of naturally occurring homologs, which often exhibit confined structural diversity, flexible backbone approaches enhance the sequence diversity, adding the challenge of identifying energetically favorable sequence variants that are correctly coupled to structural perturbations [10].

Another variation for computational design approaches i*s de novo* design, in which protein backbones are assembled *in silico*, followed by sequence optimization to fold into a pre-defined three-dimensional conformation without being constrained by previous sequence information [11–13]. This approach tests our understanding of the rules governing the structure of different protein folds. The failures and successes of this approach confirm and correct the principles used for the protein design process [7, 8].

One of the main aims of computational protein design is the rational design of functional proteins capable of carrying existing or novel functions into new structural contexts [14]. Broadly, there are three main approaches for the design of functional proteins: redesigning of pre-existing functions, grafting of functional sites onto heterologous proteins, and designing of novel functions not found in the protein repertoire. The redesign of a pre-existing function to alter its catalytic activity [15] or improve its binding target recognition [16] can be considered the most conservative approach. It is typically accomplished by point mutations around the functional area of interest and tends to have little impact on global structure of the designed protein. On the other extreme, the design of fully novel functions has most noticeably been achieved by applying chemical principles that tested our fundamental knowledge of enzyme catalysis [17, 18].

Between these two approaches resides protein grafting. This method aims to repurpose natural folds as carriers for exogenous known functions. It relies on the strong structure-function relationship present in proteins, to endow an heterologous protein with an exogenous function by means of transferring a structural motif that performs such function [3–5, 19–22].

At the biochemical level, grafting approaches have been used to design high binding affinity protein-protein interactions, by stabilizing binding motifs removing the entropic cost of binding (e.g. flexible peptides) [21], and also by extending the binding interfaces to allow for additional energetically favorable interactions. The extended interfaces also provide opportunities to tune the specificity of the designed proteins [21]. On the practical side, some of the most notable applications of protein grafting thus far, have been the design of novel viral inhibitors [21, 23] and epitope-focused immunogens for vaccine design [3–5]. Following this strategy one can easily imagine applications to functionalize protein-based biomaterials [24] or to design novel biosensors [25]. The importance of robust grafting approaches to functionalize heterologous proteins is related to the fact that the proteins that naturally perform these functions, may lack the best biochemical properties in terms of size, affinity, solubility, immunogenicity and other application specific factors.

Thus far, the most successful grafting approaches are highly dependent on structural similarity between the functional motif and the insertion region in the protein scaffold. When the functional motif and the insertion region are identical in backbone conformation, the motif transfer can be performed by side-chain grafting, i.e. mutating the target residues into those of the functional motif [3, 5]. In much more challenging scenarios, full backbone grafting may be used in conjunction with directed evolution [19]. Nevertheless, motif transfer is limited between very similar structural regions, which greatly constrains the subset of putative scaffolds that can be used for this purpose. The lack of compatibility between the putative scaffolds and the functional sites has been referred to as a “designability” problem [26, 27], which refers to the likelihood of a protein backbone to host and stabilize a structural motif. The designability problem becomes more obvious as the structural complexity of the functional motif grows, drastically limiting the types of functional motifs that can be transferred. Previously, we have demonstrated the possibility of expanding protein grafting to scaffolds with segments that have low structural similarity. To accomplish that task, we developed the prototype protocol Rosetta Fold From Loops (FFL) [4, 21].

The distinctive feature of our protocol is the coupling of the folding and design stages to bias the sampling towards structural conformations and sequences that stabilize the grafted functional motif. In the past, FFL was used to obtain designs that were functional (synthetic immunogens [4] and protein-based inhibitors [21]) and where the experimentally determined crystal structures closely resembled the computational models. However, the structures of the functional sites were structurally very close to the insertion segments of the hosting scaffolds. The architecture of FFL was intrinsically limited in the types of constraints available and the grafting of linear, single segment functional motifs.

Here, we present a complete re-implementation of FFL with enhanced functionalities, simplified user interface and complete integration with other Rosetta protocols. We have called this new, more generalist protocol Rosetta Functional Folding and Design (FunFolDes). We benchmarked FunFolDes extensively, unveiling important technical details to better exploit and expand the capabilities of the protocol. Furthermore, we challenged FunFolDes with two design tasks of transplanting viral epitopes to heterologous scaffolds, and by doing so probe the applicability of the protocol. The design tasks were centered on using distant structural templates as hosting scaffolds, and functionalizing a *de novo* designed protein,—FunFolDes succeeded in both challenges. These results are encouraging and provide a solid basis for the broad applicability of FunFolDes as a strategy for the robust computational design of functionalized proteins.

## Results

### Rosetta FunFolDes–a computational framework for design of functional proteins

The original prototype of the Rosetta Fold From Loops (FFL) protocol was successfully used to transplant the structural motif of the Respiratory Syncytial Virus protein F (RSVF) site II neutralizing epitope into a protein scaffold in the context of a vaccine design application [4].

FFL enabled the insertion and conformational stabilization of the structural motif into a defined protein topology by using Rosetta’s fragment insertion machinery to fold an extended polypeptide chain to adopt the desired topology [28] which was then sequence designed. Information from the scaffold structure was used to guide the folding, ensuring an overall similar topology while allowing for the conformational changes needed to stabilize the inserted structural motif.

**Fig 1:**
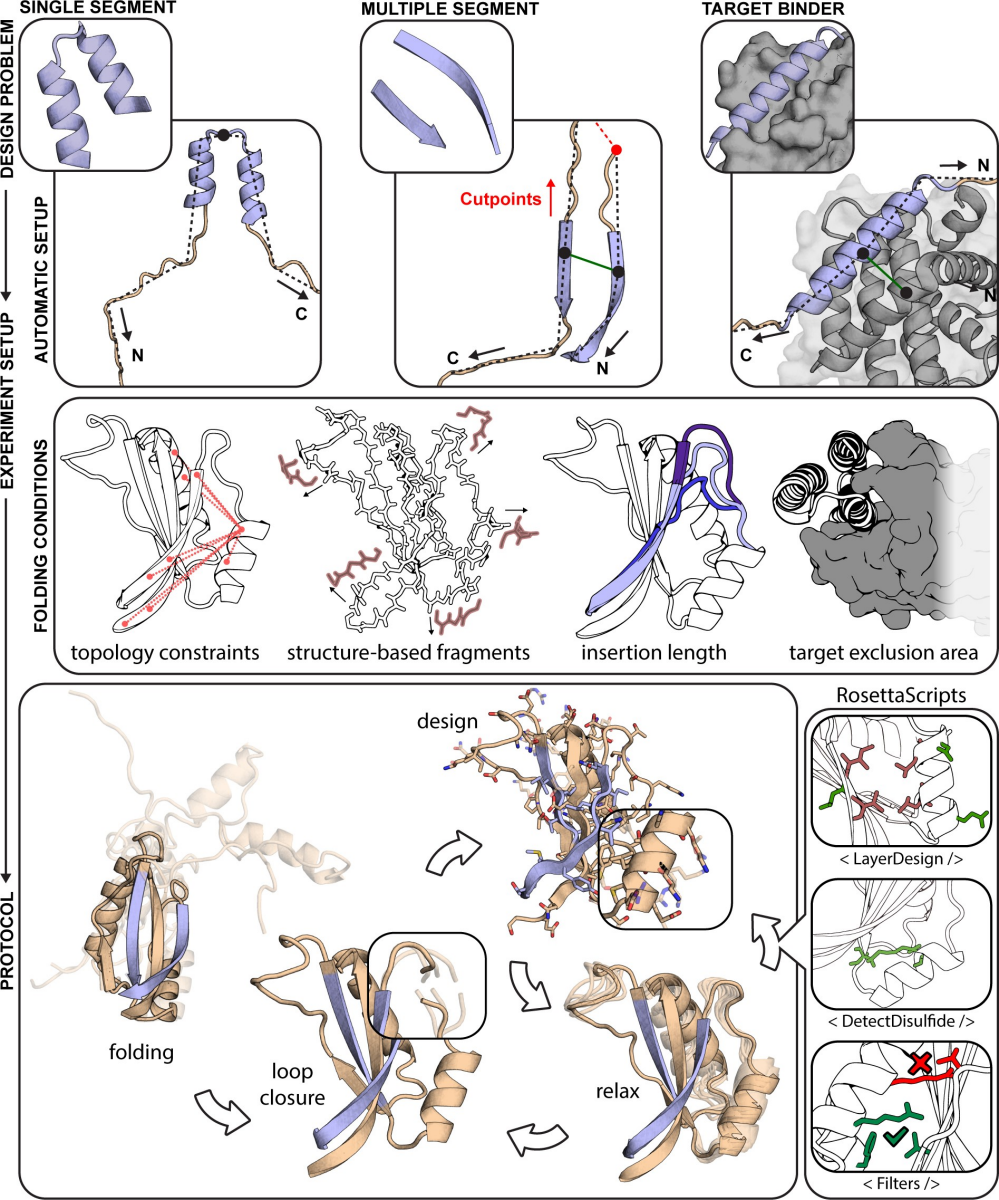
Rosetta FunFolDes—method overview. FunFolDes was devised to tackle a wide range of functional protein design problems, combining a higher user control of the simulation parameters whilst simultaneously lowering the level of expertise required. FunFolDes is able to transfer single- and multi-segment motifs (light blue) together with the target partner (grey) by exploiting Rosetta’s FoldTree framework (top row). A wider range of information can be extracted from the template (wheat) to shift the final conformation towards a more productive design space (middle row), including targeted distance constraints, generation of structure-based fragments, motif insertion in sites with different residue length and presence of the binding target to bias the folding stage. The bottom row showcases the most typical application of the FunFolDes protocol. Implementation in RosettaScripts allows to tailor FunFolDes behavior. A seamless integration with other protocols and complex selection logics can be added to address the different needs in each design task.

The final implementation of FunFolDes is schematically represented in **Fig 1**, and fully described in Materials and Methods. Our upgrades to FFL focused on three main aims: I) improve the applicability of the system to handle more complex structural motifs (i.e. multiple discontinuous backbone segments); II) enhance the design of functional proteins by including binding partners in the simulations; III) increase the control over each stage of the simulation improving the usability for non-experts. These three aims were achieved through the implementation of five core technical improvements described below.

**Insertion of multi-segment functional sites**. Most functional sites in proteins typically entail, at the structural level, multiple discontinuous segments, as is the case for protein-protein interfaces, enzyme active-sites, and others [29, 30]. FunFolDes handles functional sites with any number of discontinuous segments, ensuring the native orientations of each of the segments. These new features enhance the types of structural motifs that can be handled by FunFolDes, widening the applicability of the computational protocol.

**Structural folding and sequence design in the presence of a binding target.** Many of the functional roles of proteins in cells require physical interaction with other proteins, nucleic acids, or metabolites [31]. The inclusion of the binder has two main advantages: I) explicit representation of functional constraints to bias the designed protein towards a functional sequence space, resolving putative clashes derived from the template scaffold; II) facilitate the design of new additional contact residues (outside of the motif) that may afford enhanced affinity and/or specificity.

**Region-specific structural constraints**. FunFolDes can collect from full-template to region-specific constraints, allowing greater levels of flexibility in areas of the scaffold that can be critical for function (e.g. segments close to the interface of a target protein). The type of distance constrains used in the protocol are soft constrains with score penalties if the defined standard deviation is exceeded in the upper and lower bounds. Furthermore, FunFolDes is no longer limited to atom-pair distance constraints [32] and can incorporate other types of kinematic constraints, such as angle and dihedral constraints [33], which have been used extensively to design beta-rich topologies [8].

**On-the-fly fragment picking**. Classically, fragment libraries are generated through sequence-based predictions of secondary structure and dihedral angles that rely on external computational methods [34]. We leveraged internal functionalities in Rosetta so that FunFolDes can assemble fragment sets on-the-fly. Using this feature, we can assemble fragment sets based on the structure of the input scaffold. Sequence-based fragments remain an option, however this feature removes the need for secondary applications, boosting the usability of FunFolDes. Lastly, the on-the-fly fragment picking enables the development of protocols with mutable fragment sets along the procedure.

**Compatibility with other Rosetta modules.** Finally, FunFolDes is compatible with Rosetta’s modular xml-interface—Rosetta Scripts (RS) [35]. Enabling customization of the FunFolDes protocol and, more importantly, cross-talk with other protocols and filters available through the RS interface.

We devised two benchmark scenarios to test the performance of FunFolDes. One of these aimed to capture conformational changes in small protein domains caused by sequence insertions or deletions, and the second scenario assessed protocol performance to fold and design a binder in the presence of the binding target.

### Capturing conformational and sequence changes in small protein domains

Typical protein design benchmarks are assembled by stripping native side chains from known protein structures and evaluating the sequence recovery of the design algorithm [9]. The main design aim of FunFolDes is to insert structural motifs into protein folds while allowing flexibility across the overall structure. This conformational freedom allows the full protein scaffold to adapt and stabilize the functional motif’s conformation. This is a main distinctive point from other approaches to design functional proteins that rely on a mostly rigid scaffold [2, 3, 11, 19, 30, 36]. For many modeling problems, such as protein structure prediction, protein-protein and protein-ligand docking, and protein design, standardized benchmark datasets are available [37] or easily accessible. Devising a benchmark for designed proteins with propagating conformational changes across the structure is challenging, as we are assessing both structural accuracy as well as sequence recovery of the protocol.

**Fig 2:**
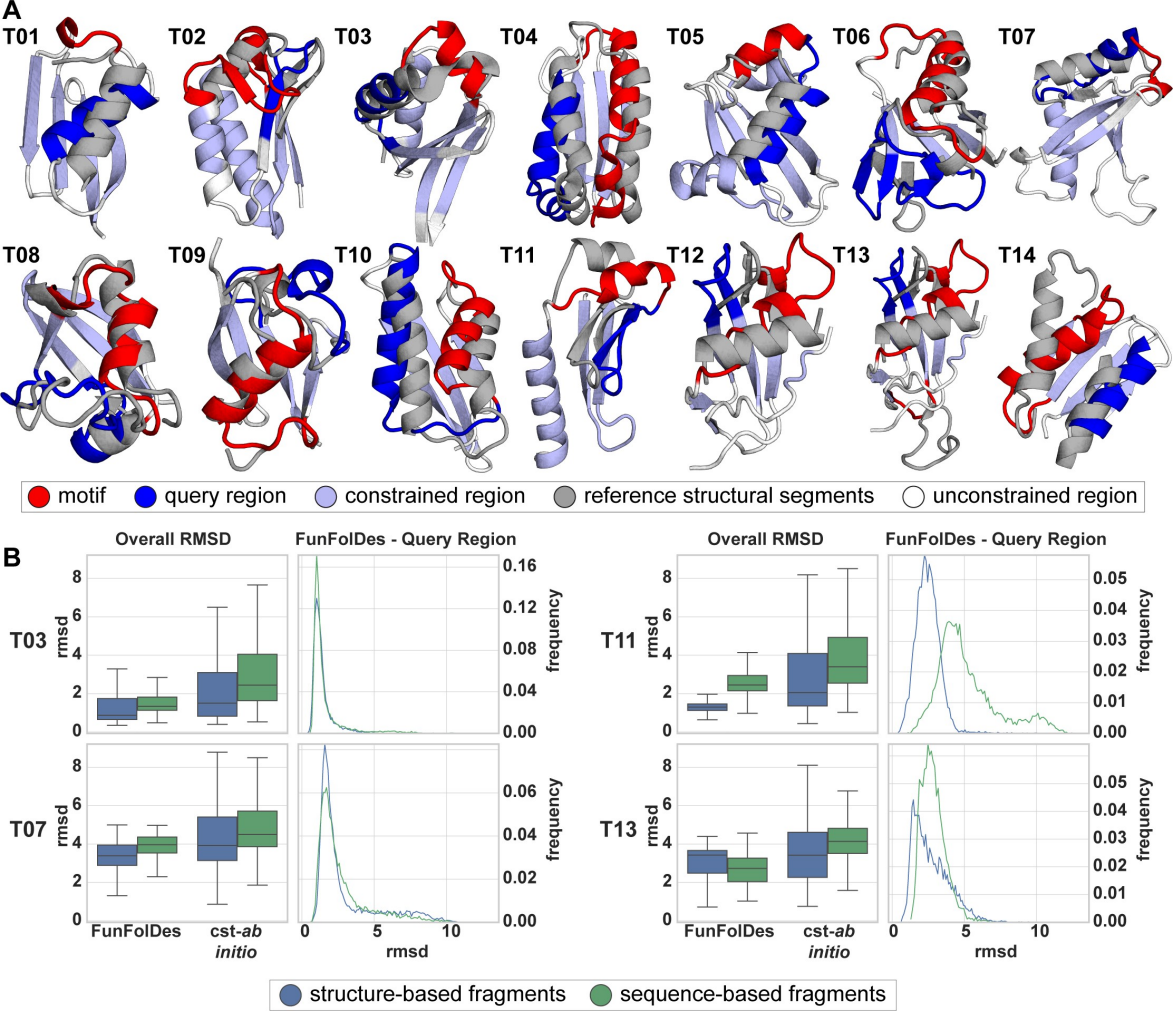
Benchmark test set to evaluate FunFolDes structural sampling. A) Structural representation of the 14 targets used in the benchmark. In each target is highlighted the motif (red) and query (blue) regions, and the positions from which distance constraints were generated (light blue). Conformations of the motif and query regions, as found in the template structures, are shown superimposed in light grey. B) Full structure RMSD (Overall RMSD) and local RMSD for the query region (FunFolDes–Query Region) is presented for four targets (full dataset presented in **S1 Fig**). Overall RMSD compares results for the two simulation modes (FunFolDes Vs. constrained–*ab initio (cst-ab initio*)) and the two fragment generation methods (structure (blue) Vs. sequence-based fragments (green)) against their original target. FunFolDes more frequently samples RMSDs closer to the conformation of the target structure. Generally, structure-based fragments contribute to lower mean overall RMSDs. The FunFolDes–Query Region RMSD distributions show that the two fragment sets do not have a major importance in the structural recovery of the query region.

To address this problem, we analyzed structural domains found repeatedly in natural proteins and clustered them according to their definition in the CATH database [38]. As a result, we selected a set of 14 benchmark targets labeled T01 through T14 (**Fig 2A**). A detailed description on the construction of the benchmark can be found in the Materials and Methods section.

Briefly, for the benchmark we selected proteins with less than 100 residues, where each test case was composed of two proteins of the same CATH domain cluster. One of the proteins is the template, and serves as a structural representative of the CATH domain. The second protein, dubbed target, contains structural insertions or deletions (motif region), to which a structural change in a different segment of the protein could be attributed (query region). The motif and query regions for all the targets are shown in **Fig 2A** and quantified by the percentage of overall secondary structure in **S1A Fig**. To a great extent, these structural changes due to natural sequence insertions and deletions are analogous to those occurring in the design scenarios for which FunFolDes was conceived.

Using FunFolDes, we folded and designed the target proteins while maintaining the motif segment structurally fixed, mimicking a structural motif insertion. Distance constraints between residues were extracted from the template in the regions of shared structural elements of the template and the target, and were used to guide the folding simulations.

To check whether FunFolDes enhances sequence and structural sampling, we compared the simulations to constrained *ab initio* (cst-*ab initio*) simulations [33]. As Rosetta conformational sampling is highly dependent upon the fragment set [39], in this benchmark we also tested the influence of structure- and sequence-based fragments. The performance of the two protocols was analyzed regarding global and local recovery of both structure and sequence.

Structural recovery was assessed through two main metrics: (a) global RMSD of the full decoys against the target and (b) local RMSD of the query region. When evaluating the distributions for global RMSD in the designed ensembles, FunFolDes outperformed cst-*ab initio* by consistently producing populations of decoys with lower mean RMSD (mostly found below 5 Å), a result observed in all 14 targets (**Fig 2B**, **S1B Fig**). This result is especially reassuring considering that FunFolDes simulations contain more structural information of the target topology than the cst-*ab initio* simulations.

The local RMSDs of the query unconstrained regions presented less clear results across the benchmark (**S1B Fig**). In 13 targets, FunFolDes outperformed cst-*ab initio*, showing lower mean RMSDs but in some targets with minor differences.

When comparing fragment sets (structure- vs sequence-based), both achieved similar mean RMSDs in the decoy populations; nonetheless, the structure-based fragments more often reached the lowest RMSDs for overall and query RMSDs (**Fig 2B**, **S1 Fig**). This is consistent with what would be expected from the structural information content within each fragment set. When paired with the technical simplicity of use, time-saving and enhanced sampling of the desired topology, the structure-based fragments are an added value for FunFolDes.

**Fig 3:**
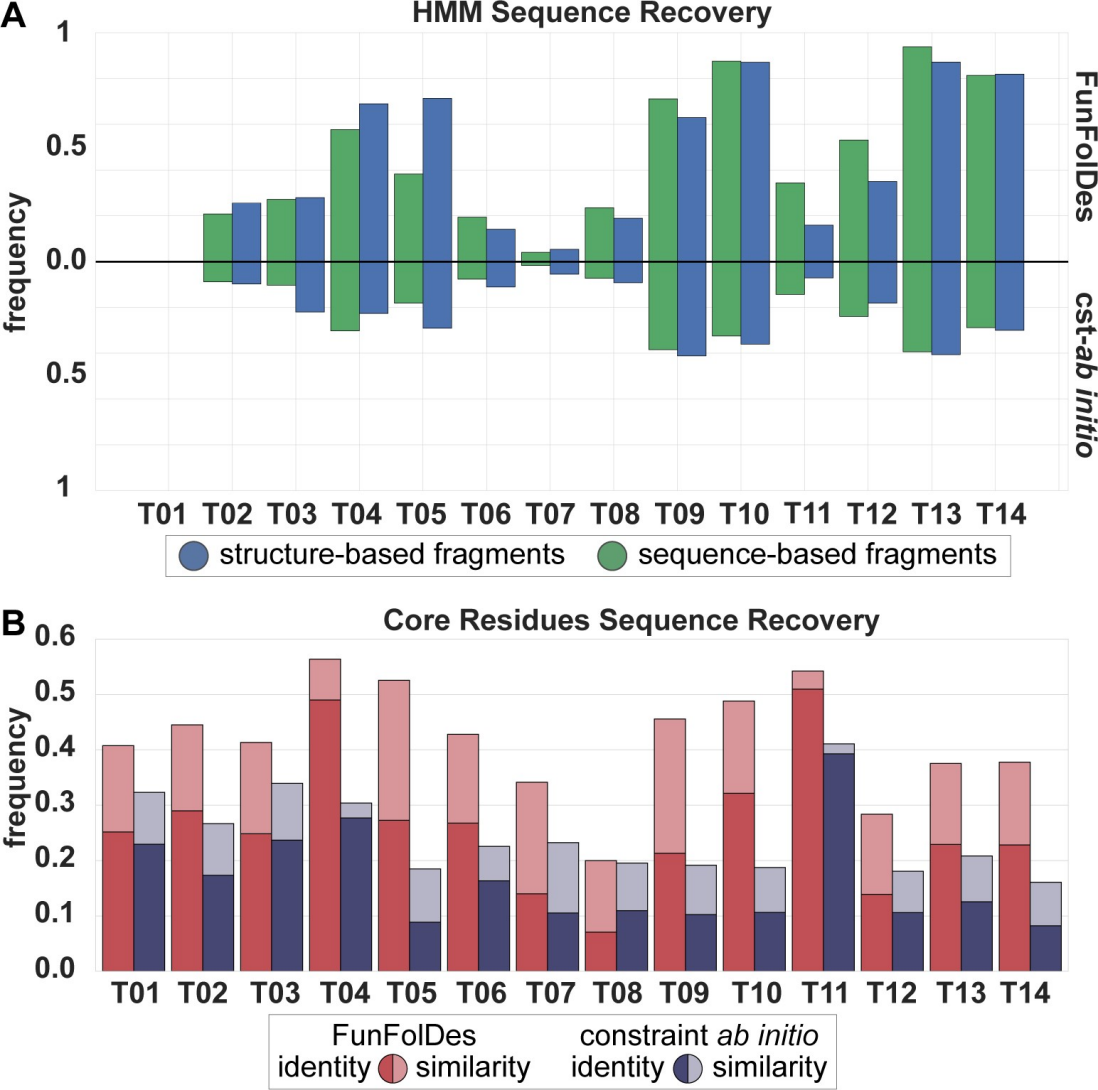
Assessment of FunFolDes sequence sampling quality. A) HMM Sequence Recovery measures the percentage of decoys generated that can be assigned to the original HMM from the CATH superfamily. FunFolDes consistently outperforms cst–*ab initio*, in agreement with the structural recovery metrics. B) Core Residues Sequence Recovery shows the sequence recovery between the core residues of the designs set and the target. Recovery is measured in terms of sequence identity and sequence similarity (as assigned through BLOSUM62). Core sequence identity and similarity was assessed over the structure-based fragment set. According to this metric FunFolDes outperforms cst-*ab initio* in every instance, reaching for some populations, levels of conservation similar to those found in more restrained flexible-backbone design approaches [10].

We also quantified sequence recovery, both in terms of sequence identity and similarity according to the BLOSUM62 matrix [40] (**Fig 3A**). In all targets, FunFolDes showed superior recoveries than cst-*ab initio*, and at the levels of other design protocols using Rosetta [10] (**Fig 3A**). This type of metrics has been shown to be highly dependent on the exact backbone conformation used as input [9, 10]. Given that FunFolDes is exploring larger conformational spaces, as a proxy for the quality of the sequences generated, we used the target’s Hidden Markov Models (HMM) [41] and quantified the designed sequences that were identified as part of the target’s CATH superfamily according to its HMM definition (**Fig 3B**). FunFolDes decoy populations systematically outperformed those from cst-*ab initio* (**Fig 3B**). The performance of the two fragment sets shows no significant differences.

In summary, this benchmark highlights the ability of FunFolDes to generate close-to-native scaffold proteins to stabilize inserted structural motifs. FunFolDes aims to refit protein scaffolds towards the structural requirements of a functional motif. It is thus critical, to explore within certain topological boundaries, structural variations around the original templates. This benchmark points to several variables in the protocol that resulted in enhanced structural and sequence sampling.

### Target-biased folding and design of protein binders

The computational design of proteins that can bind with high affinity and specificity to targets of interest remains a largely unsolved problem [42]. Within the FunFolDes conceptual approach of coupling folding with sequence design, we sought to add the structure of the binding target (**Fig 1**) to attempt to bias sampling towards functional structural and sequence spaces.

**Fig 4:**
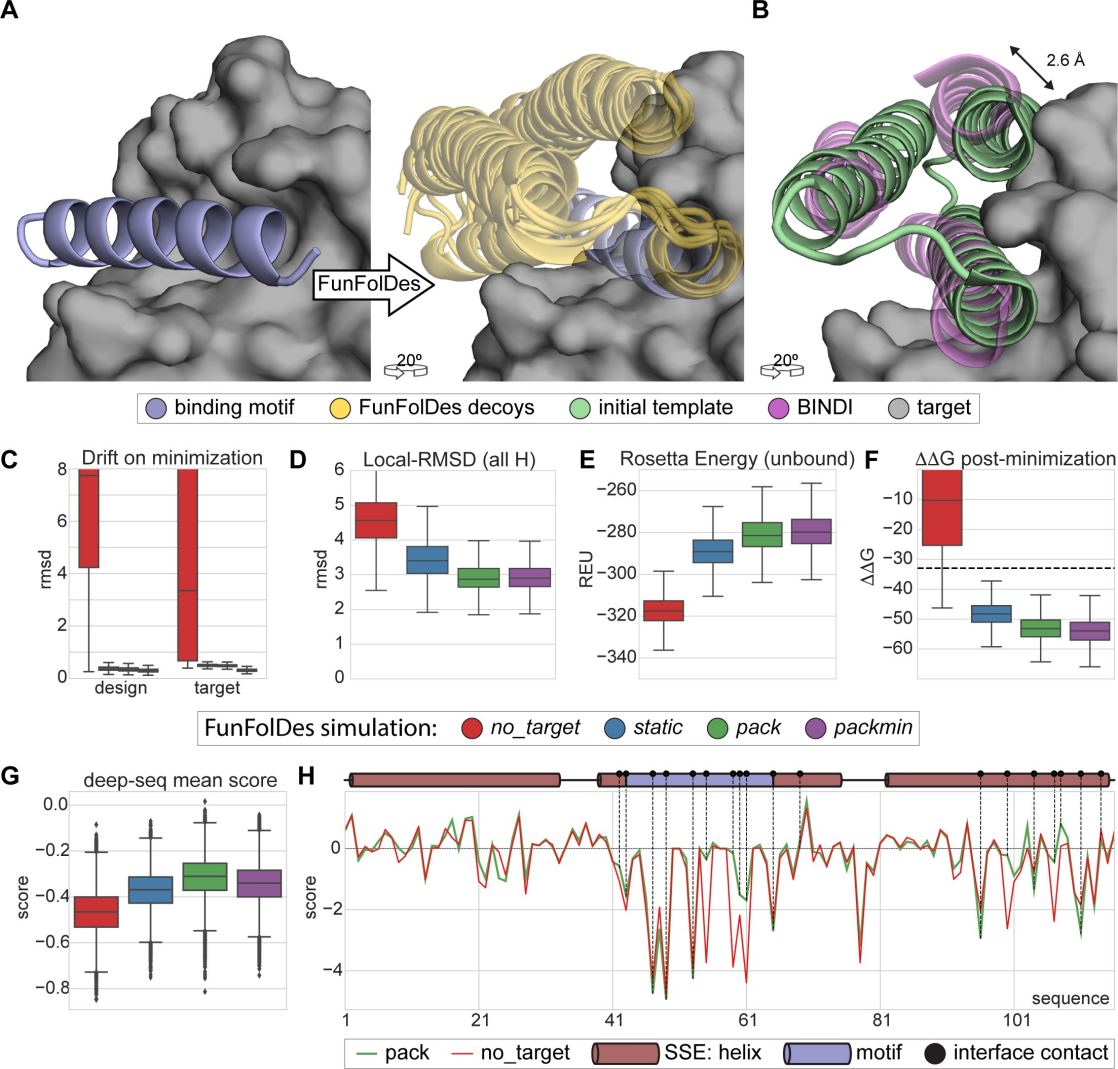
Target-biased design of a protein binder and performance assessment based on saturation mutagenesis. A) Depiction of the initial design task, a single-segment binding motif (BIM-BH3) shown in light blue cartoons, with its target (BHRF1) shown in gray surface, is used by FunFolDes to generate an ensemble of designs compatible with the binding mode shown in light orange cartoons. B) Conformational difference between the initial template (PDB ID: 3LHP), shown in light brown and the previously designed binder (BINDI), shown in violet cartoons, helix 3 requires a subtle but necessary shift (2.6 Å) to avoid steric clashes with the target. C-G) Scoring metrics for design populations according to the simulation mode: *no_target*—FunFolDes was used without the target protein; *static*—target present no flexibility allowed; *pack*—target allowed to repack the side-chains; *packmin*–side-chain repacking plus minimization and backbone minimization were allowed for the target. The target flexibility was allowed during the relax-design cycles of FunFolDes. C) Structural drift observed for design and target binder measured as the RMSD between pre- and post-minimization conformations. D) Structural recovery of the conformation observed in the BINDI-BHRF1 assessed over the 3 helical segments of the bundle. E) Rosetta energy for the designs in the unbound state generated by different simulation modes. F) Interaction energy (ΔΔG) between the designs and the target. G) Deep-sequencing score distribution for each design population, computed as the mean score of each sequence after applying a position score matrix based on the deep-sequencing data. The *pack* population slightly outperforms the other simulation modes. H) Per-residue scoring comparison of the *no_target* and the *pack* populations according to the deep-sequencing data. Although the behavior is overall similar, *pack* outperforms *no_target* in multiple positions, several of which are highlighted (black dots) as interfacial contacts or second shell residues close to the binding site.

Previously, we used FFL to design a new binder (BINDI) to BHRF1 (**Fig 4A**), an Epstein-Barr virus protein with anti-apoptotic properties directly linked to the tumorigenic activity of EBV [21]. FFL designs bound to BHRF1 with a dissociation constant (*K*_D_) of 58–60 nM, and after affinity maturation reached a *K*_D_ of 220±50 pM. BINDI was designed in the absence of the target and then docked to BHRF1 through the known interaction motif. A striking observation from the overall approach was that the FFL stage was highly inefficient, generating a large fraction of backbone conformations incompatible with the binding mode of the complex.

To test whether the presence of the target could improve structural and sequence sampling, we leveraged the structural and sequence information available for the BINDI-BHRF1 system and benchmarked FunFolDes for this design problem. As described by Procko and colleagues, when comparing the topological template provided to FFL and the BINDI crystal structure, the last helix of the bundle (helix 3) was shifted relative to the template ensuring structural compatibility between BINDI and BHRF1 (**Fig 4B**). We used this case study to assess the capabilities of FunFolDes to sample closer conformations to those observed in the BINDI-BHRF1 crystal structure. In addition, we used the saturation mutagenesis data generated for BINDI [21] to evaluate the sequence space sampled by FunFolDes.

A detailed description of this benchmark can be found in the Materials and Methods section. Briefly, we performed four different FunFolDes simulations: I) binding target absent (*no_target*); II) binding target present with no conformational freedom (*static*); III) binding target present with side-chain repacking (*pack*); IV) binding target present with side-chain repacking plus minimization and backbone minimization (*packmin*).

*no_target* simulations generated a low number of conformations compatible with the target (<10% of the total generated designs) (**S2A Fig**). Upon global minimization more than 60% (**S2A Fig**) of the decoys were compatible with the binding target, at the cost of considerable structural drifts for both binder (mean RMSD 3.3 Å) and target (mean RMSD 7.7 Å) (**Fig 4C**). These structural drifts reflect the energy optimization requirements by the relaxation algorithms but are deemed biologically irrelevant due to the profound structural reconfigurations. In contrast, simulations performed in the presence of the target clearly biased the sampling to more productive conformational spaces. RMSD drifts upon minimization were less than 1 Å for both designs and binding target (**Fig 4C**).

Global structural alignments of the designs fail to emphasize the differences of the helical arrangements (**S2B Fig**). Thus, we aligned all the designs on the conserved binding motif (**Fig 4A**) and measured the RMSD over the three helices that compose the fold. FunFolDes simulations in the presence of the target sampled a mean RMSD of 3 Å (lowest ≈ 2 Å) compared to the BINDI structure (**Fig 4D**), with the closest designs at approximately 2 Å, while the *no_target* simulation showed a mean RMSD of 4.5 Å (lowest ≈ 2.5 Å). While we acknowledge that these structural differences are modest, the data suggests that they can be important to sample conformations and sequences competent for binding.

We also analyzed Rosetta energy distributions of designs in the unbound state for the different simulations. We observed noticeable differences for the designs generated in the absence (*no_target*) and the presence of the binding target, -320 and -280 Rosetta Energy Units (REUs), respectively (**Fig 4E**). This difference is significant, particularly for a small protein (116 residues). We also observed considerable differences for the binding energies (ΔΔG) of the *no_target* and the bound simulations with mean ΔΔGs of -10 and -50 REUs, respectively (**Fig 4E**).

The energy metrics provide interesting insights regarding the design of functional proteins. Although the sequence and structure optimization for the designs in the absence of the target reached lower energies, these designs are structurally incompatible with the binding target and, even after refinement, their functional potential (as assessed by the ΔΔG) is not nearly as favorable as those performed in the presence of the binding target (**Fig 4F**). These data suggest that, in many cases, to optimize function it may be necessary to sacrifice the overall computed energy of the protein, a common proxy to the experimental thermodynamic stability of the protein [43]. The existence of stability-function tradeoffs has been the subject of many experimental studies [44, 45], however, it remains a much less explored strategy in computational design, where it may also be necessary to design proteins with lower stability to ensure that the functional requirements can be accommodated. This observation provides a compelling argument to perform biased simulations in the presence of the binding target, which can be broadly defined as a “functional constraint”.

To evaluate sequence sampling quality, we compared the computationally designed sequences to a saturation mutagenesis dataset available for BINDI [21].

The details of the dataset and scoring scheme can be found in the methods and S2 Fig. Briefly, point mutations beneficial to the binding affinity to BHRF1 have a positive score, deleterious mutants a negative score, and neutral score 0. Such a scoring scheme, will yield a score of 0 for the BINDI sequence.

Designs performed in the presence of the binding target obtained higher mean scores as compared to the *no_target* designs (**Fig 4G**). The *pack* simulation, showed the highest distribution mean, having one design scoring better than the BINDI sequence. In some key positions at the protein-protein interface, the *pack* designs clearly outperformed those generated by the *no_target* simulation, when quantified by a per-position score (**Fig 4H**); meaning that amino-acids productive for binding interactions were sampled more often. This benchmark provides an example of the benefits of using a “functional constraint” (binding target) to improve the quality of the sequences obtained by computational design.

Overall, the BINDI benchmark provided important insights regarding the best FunFolDes protocol to improve the design of functional proteins.

### Repurposing a naturally occurring fold for a new function

**Fig 5:**
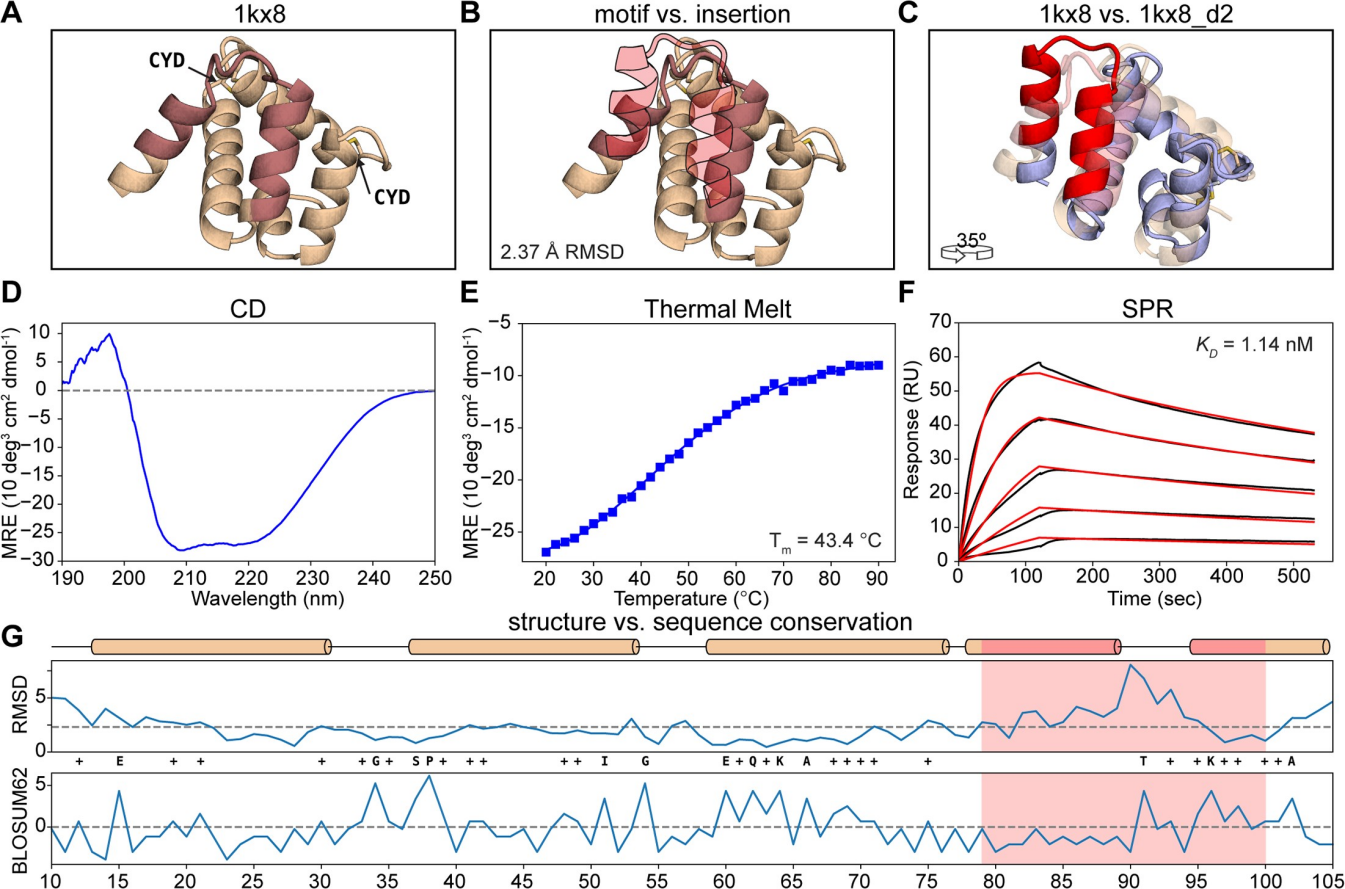
Functional design of a distant structural template. A) Structural representation of 1kx8. The insertion region is colored in light red and the two disulfide bonds are labeled (CYD). B) Structural comparison between the insertion region of 1kx8 and the site II epitope (light red-filled silhouette). The local RMSD between the two segments is 2.37 Å. C) Superposition between 1kx8_d2 design model (blue with red motif) and the 1kx8 template (wheat and light red insertion site). Multiple conformational shifts are required throughout the structure to accommodate the site II epitope. D) CD spectrum of 1kx8_d2 showing a typical alpha-helical pattern with the ellipticity minima at 208 nm and 220 nm. E) 1kx8_d2 shows a melting temperature (T_m_) of 43.4°C. F) Binding affinity determined by SPR. 1kx8_d2 shows a *K*_D_ of 1.14 nM. Experimental sensorgrams are shown in black and the fitted curves in red. G) Per-position evaluation of structural (top) and sequence (bottom) divergence between the design model 1kx8_d2 and the starting template 1kx8. The largest structural differences are observed in the epitope insertion region, the overall difference of the two structures is 2.25 Å (dashed line). The sequence was evaluated using the BLOSUM62 score matrix, yielding a total of 13.5% identity and 38.5% similarity. The epitope region is colored in light red. Identical positions between the 1kx8_d2 and 1kx8 are labeled with the residue one letter code, while positively scored changes are labeled with plus (+).

To further test FunFolDes’s design capabilities, we sought to transplant a contiguous viral epitope that is recognized by a monoclonal antibody with high affinity (**Fig 5A**). For this design, we used the RSVF site II epitope (PDB ID: 3IXT [46]) as the functional motif. This epitope adopts a helix-loop-helix conformation recognized by the antibody motavizumab (mota) [46]. In previous work we have designed proteins with this epitope, but started from a structurally similar template, where the RMSD between the epitope and the scaffold segment was approximately 1 Å over the helical residues. Here, we sought to challenge FunFolDes by using a distant structural template where the local RMSDs of the epitope and the segment onto which it was transplanted were higher than 2 Å. We used MASTER [47] to perform the structural search (detailed description in Materials and Methods) and selected as template scaffold the structure of the A6 protein of the Antennal Chemosensory system from the moth *Mamestra brassicae* (PDB ID: 1KX8 [48])(**Fig 5A**). The backbone RMSD between the conformation of the epitope and the insertion region in 1kx8 is 2.37 Å (**Fig 5B**).

The A6 protein is involved in chemical communication and has been shown to bind to fatty-acid molecules with hydrophobic alkyl chains composed of 12–18 carbons. Two prominent features are noticeable in the structure: two disulfide bonds (**Fig 5A**) and a considerable void volume in the protein core (**S3 Fig**), thought to be the binding site for fatty acids. These features emphasize that the initial design template is likely not a very stable protein.

In the design process we performed two stages of FunFolDes simulations to obtain a proper insertion of the motif in the topology (**Fig 5C**). A detailed description of the workflow and metrics used for selection (**S3 Fig**) can be found in the Materials and Methods. A striking feature of our designs, when compared to the starting template, is that they had a much lower void volume, showing that FunFolDes generated structures and sequences that yielded well packed structures (**S3 Fig**).

We started by testing experimentally seven designs. Those that expressed in bacteria were further characterized using size exclusion chromatography coupled to a multi-angle light scatter (SEC-MALS) to determine the solution oligomerization state. To assess their folding and thermal stability (T_m_) we used Circular Dichroism (CD) spectroscopy, and finally to assess their functional properties we used surface plasmon resonance (SPR) to determine binding dissociation constants (*K*_*D*_s) to the mota antibody. Out of the seven designs, six were purified and characterized further. The majority of the designs were monomers in solution and showed CD spectra typical of helical proteins. Regarding, thermal stabilities we obtained designs that were not very stable and did not unfold cooperatively (1kx8_02), however we also obtained very stable designs that did not fully unfold under high temperatures (1kx8_07) (**S4 Fig**).

The determined binding affinities to mota ranged from 34 to 208 nM, which was an encouraging result. Nevertheless, compared to the peptide epitope (*K*_*D*_ = 20 nM) and other designs previously published (*K*_*D*_ = 20 pM) [4], there was room for improvement. Therefore, we generated a second round of designs to attempt to improve stability and binding affinities.

Driven by the observation that the native fold has two disulfide bonds, in the second round, we tested eight designed variants with different disulfide bonds and, if necessary, additional mutations to accommodate them. The disulfide bonded positions were selected according to the spatial orientation of residues in the designed models, with most of the disulfide bonds being placed at distal locations from the epitope (>20 Å). All eight designs were soluble after purification and two were monomeric: 1kx8_d2 and 1kx8_3_d1, showing CD spectra typical of helical proteins (**Fig 5D**) with melting temperatures (T_m_s) of 43 and 48°C (**Fig 5E**), respectively. Remarkably, 1kx8_d2 showed a *K*_*D*_ of 1.14 nM (**Fig 5F**), an improvement of approximately 30-fold compared to the best variants of the first round. 1kx8_d2 binds to mota with approximately 20-fold higher affinity than the peptide-epitope (*K*_*D*_ ≈ 20 nM), and 50-fold lower compared to previously designed scaffolds (*K*_*D*_ = 20 pM) [4]. This difference in binding is likely reflective of how challenging it can be to accomplish the repurposing of protein structures with distant structural similarity.

Post-design analyses were performed to compare the sequence and structure of the best design model with the initial template. The global RMSD between the two structures is 2.25 Å. Much of the structural variability arises from the inserted motif, while the surrounding segments adopt a configuration similar to the original template scaffold. The sequence identity of 1kx8_d2 as compared to the native protein is approximately 13%. The sequence conservation per-position (**Fig 5G**) was evaluated through the BLOSUM62 matrix, where positive scores are attributed to the original residue or favorable substitutions and negative if unfavorable. Overall, 38.5% of the residues in 1kx8_d2 scored positively, and 61.4% of the residues had a score equal or lower than 0. This is particularly interesting, from the perspective that several residues, unfavorable according to BLOSUM62, yielded well folded and functional proteins. To further substantiate our experimental results, we performed structure prediction simulations of the designed sequences, where we observed that 1kx8_d2 presents a higher folding propensity than the WT protein (S5A Fig). To evaluate if the predicted models presented the correct epitope conformation, we performed docking simulations and observed that they obtained lower binding energies than the native peptide-antibody complex, within similar RMSD fluctuations (S5A Fig).

The successful design of this protein is a relevant demonstration of the broad usability of FunFolDes and the overall strategy of designing functional proteins by coupled folding and design to incorporate functional motifs in unrelated protein scaffolds.

### Functionalization of a functionless fold

**Fig 6:**
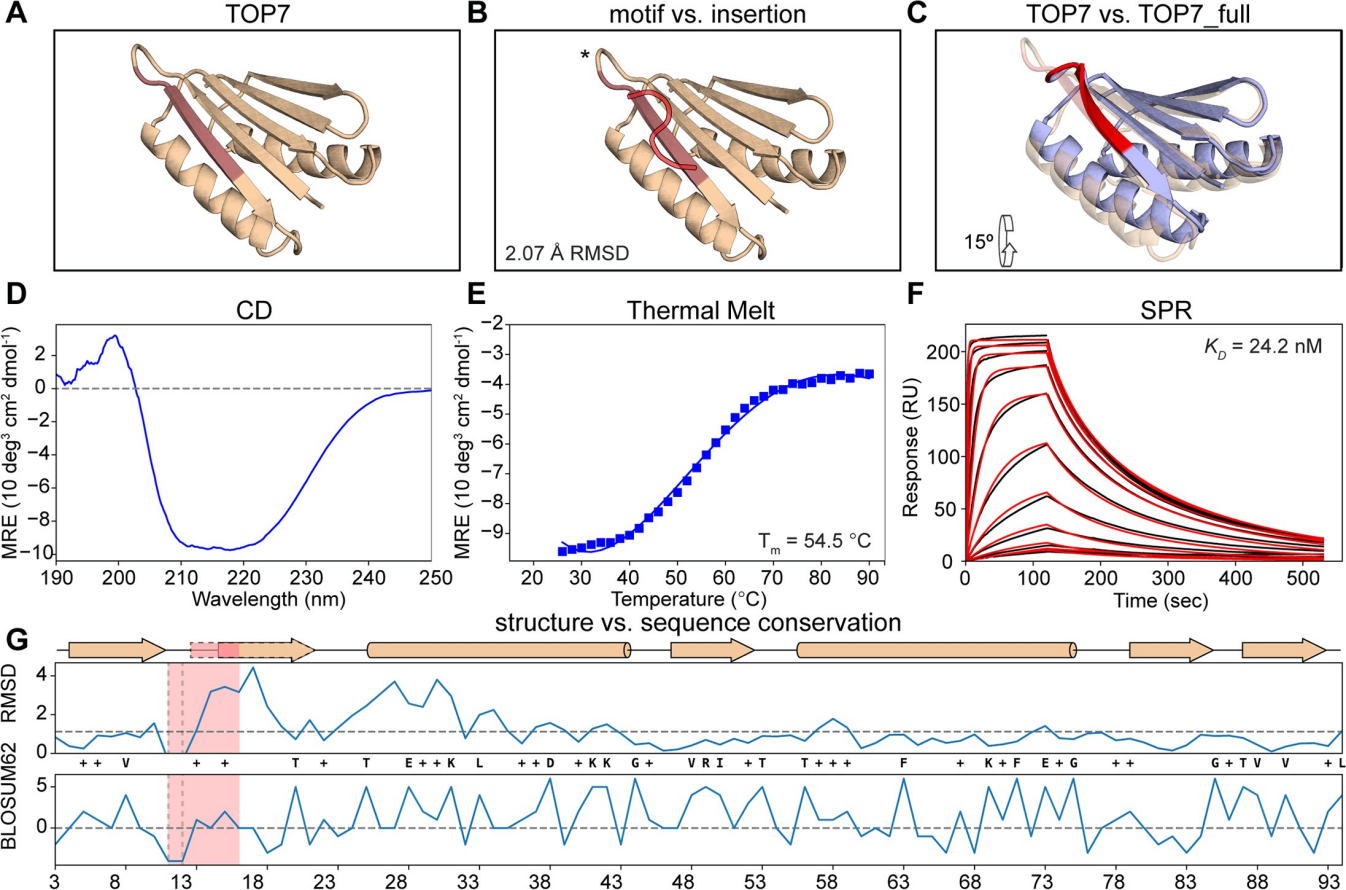
Functionalization of the functionless de novo fold TOP7. A) Structure of TOP7 with the insertion region highlighted in light red. B) Structural comparison between 101F and TOP7’s insertion region shows a 2.1 Å RMSD. C) TOP7_full model (in blue and red for the motif) superimposed over the TOP7 crystal structure. 101F’s insertion is structurally compensated mostly by the first pairing beta strand and a shift of the first alpha helix. D) CD spectrum shows a broad ellipticity signal between 210 nm and 222 nm as a representative of mixed alpha and beta secondary structures. E) The T_m_ for TOP7_full was 54.5°C. F) Binding affinity determined by SPR. TOP7_full shows a *K*_*D*_ of 24.2 nM. Experimental sensorgrams are shown in black and the fitted curves in red. G) Per-position evaluation of structural (top) and sequence (bottom) divergence between the design model TOP7_full and the starting template TOP7. The largest structural differences are observed in the region downstream of the site IV epitope, the overall difference of the two structures is 1.5 Å (dashed horizontal line). The connecting loop between the strand that holds the epitope and the adjacent strand was also shortened to obtain a tighter connection between the 2 strands (dashed vertical region). Sequence divergence is evaluated by applying the BLOSUM62 score matrix to the sequences, yielding a total of 27.7% identity and 52.2% similarity. The epitope region is colored in light red. Identical positions between the TOP7_full and TOP7 are displayed as their residue types while positively scored changes according to BLOSUM62 are labeled with a plus (+).

Advances in computational design methodologies have achieved remarkable results in the design of *de novo* protein sequences and structures [7, 8, 11]. However, the majority of the designed proteins are “functionless” and were designed to test the performance of computational algorithms for structural accuracy. Here, we sought to use one of the hallmark proteins from *de novo* design efforts–TOP7 [13] (**Fig 6A**)–and functionalize it using FunFolDes. The functional site selected to insert into TOP7 was a different viral epitope from RSVF, site IV, which is recognized by the 101F antibody [49]. When bound to the 101F antibody, site IV adopts a β-strand-like conformation (**Fig 6B**), which in terms of secondary structure content is compatible with one of the edge strands of the TOP7 topology (**Fig 6C**). Despite the secondary structure similarity, the RMSD of the site IV backbone in comparison with TOP7 is 2.1 Å over 7 residues, and the antibody orientation in this particular alignment reveals steric clashes with TOP7. Therefore, this design challenge is yet another prototypical application for FunFolDes, and we followed two distinct design routes: I) a conservative approach where we fixed the amino-acid identities of roughly half of the core of TOP7 and allowed mutations mostly on the contacting shell of the epitope insertion site; and II) a sequence unconstrained design where all the positions of the scaffold were allowed to mutate. We attempted five designs for recombinant expression in *E*. *coli* and two (TOP7_full and TOP7_partial) were selected for further biochemical and biophysical characterization, one from each of the two design strategies mentioned above. According to SEC-MALS, both behaved as monomers in solution, with TOP7_partial showing higher aggregation propensity. Both TOP7_full and TOP7_partial (**S6 Fig**) were folded according to CD measurements. TOP7_full showed a CD spectrum (**Fig 6D**) very similar to that of native TOP7 [13]. We observed that TOP7_full was much less stable than the original TOP7 (**Fig 6E**)(T_m_ = 54.5°C). To quantify the functional component of TOP7_full, we determined a *K*_*D*_ of 24.2 nM with 101F (**Fig 6F**), within the range measured for the native viral protein RSVF (3.6 nM) [49]. Importantly, the *K*_*D*_ for TOP7_full is 2400 fold lower than that of the peptide-epitope (58.4 μM) [49], suggesting that productive conformational stabilization and/or extra contacts to the scaffold were successfully designed.

Per-residue structural similarity and sequence recovery were evaluated for TOP7_full against TOP7 (**Fig 6G**). Most conformational changes occur on the site IV insertion region and displacement of the neighboring alpha-helix, with the overall backbone RMSD being 1.5 Å.

Remarkably, the sequence identity of the most aggressive design (TOP7_full) is only 28%, and using the BLOSUM62 based scoring system, we observe that most of the TOP7_full residues were actually favorable, obtaining positive scores. This low conservation is especially relevant considering that intensive studies on TOP7 have revealed the importance of beta-sheet conservation in order to keep its foldability [22, 50, 51]. Sequence folding prediction experiments showed that TOP7_full has a similar folding propensity to TOP7 and docking simulations also show lower binding energies as compared to the native peptide-antibody complex, reinforcing the experimental results obtained (S5B Fig),

In summary, our results show that FunFolDes repurposed a functionless protein by folding and designing its structure to harbor a functional site, which in this case was a viral epitope. Previously, these computationally designed proteins with embedded viral epitopes were dubbed epitope-scaffolds and showed their medical applicability as immunogens that elicited viral neutralizing antibodies [4].

## Discussion

The robust computational design of proteins that bear a biochemical function remains an important challenge for current methodologies. The ability to consistently repurpose old folds for new functions or the *de novo* design of functional proteins could bring new insights into the determinants necessary to encode function into proteins (e.g. dynamics, stability, etc.), as well as, important advances in translational applications (e.g. biotechnology, biomedical, biomaterials, etc.).

Here, we present Rosetta FunFolDes, that was conceived to embed functional motifs into protein topologies. This protocol allows for a global retrofitting of the overall protein topology to favorably host the functional motif and enhance the designability of the starting structural templates. FunFolDes has evolved to incorporate two types of constraints to guide the design process: topological and functional. The former entails the fragments to assemble the protein structure and sets of spatial constraints that bias the folding trajectories towards a desired topology; and the latter are the structure of the functional motif and the binding target.

Our methodological approach fills the gap between conservative grafting approaches where the structure of the host scaffold is mostly fixed (Rosetta Epigraft and Motifgraft [19, 52]) and the full *de novo* assembly of non-predefined protein topologies bearing functional motifs [53, 54]. FunFolDes lies in between, by affording considerable structural flexibility to the host scaffold within the boundaries of its topology. In our view, FunFolDes is the most appropriate tool in situations where the structural mimicry of the functional motif is distant from the receiving scaffold’s site and overall conformational adaptations are necessary to design viable protein structures and sequences.

We have extensively benchmarked FunFolDes, leveraging natural structural and sequence variation of proteins within the same fold, as well as deep mutational scanning data for the computationally designed protein BINDI [21]. In our first benchmark, we observed that FunFolDes biases the sampling towards improved structural and sequence spaces. Improved sampling may contribute to solve some of the major limitations in protein design, related to “junk” sampling, where many designs are not physically realistic, exhibiting flaws according to general principles of protein structure. Importantly, higher quality sampling will likely contribute to improve the success rate of designs that are tested experimentally. The BINDI benchmark allowed us to test FunFolDes in a system with extensive experimental data, which included both sequences and structures. Perhaps the most interesting observation was that designs that were theoretically within a sequence/structure space productive for binding, were far from the energetic minimum accessible to the protein fold in the absence of the binding target. This observation resembles the stability-function tradeoffs that have been reported from in vitro evolution experimental studies [44, 45]. The large majority of the design algorithms are energy “greedy” and the sequence/structure searches are performed with the central objective of finding the global minimum of the energetic landscape. By introducing functional constrains into the simulations, FunFolDes presents an alternative way of designing functional molecules and skew the searches towards off-minima regions of the global landscape. We anticipate that such finding will be more relevant for protein scaffolds that need to undergo a considerable structural adaptation to perform the desired function. If confirmed that this finding is generalized across multiple design problems, it could be an important contribution for the field of computational protein design.

Furthermore, we used FunFolDes to tackle two design challenges and functionalized two proteins with two distinct viral epitopes generating synthetic proteins that could have important translational applications in the field of vaccine development. In previous applications, FFL always used three-helix bundles as design templates, here we diversified the template folds and used an all-helical protein that is not a bundle (1kx8) and a mixed alpha-beta protein (TOP7), clearly demonstrating the applicability to other folds. For the 1kx8 design series, we evaluated the capability of using distant structural templates as starting topologies as a demonstration of how to functionally repurpose many naturally occurring protein structures available. We obtained stable proteins that were recognized by an anti-RSV antibody with high affinity, showing that in this case, we successfully repurposed a distant structural template for a different function, a task for which other computational approaches [55] would have limited applicability. We see this result as an exciting step forward towards using the wealth of the natural structural repertoire for the design of novel functional proteins.

In a last effort, we functionalized a “functionless” fold, based on one of the first *de novo* designed proteins–TOP7. For us, this challenge has important implications to understand the design determinants and biochemical consequences of inserting a functional motif into a protein that was mainly optimized for thermodynamic stability. We were successful in functionalizing TOP7 differently than previous published efforts. Previously, TOP7 was mostly used as a carrier protein with functional motifs fused onto loop regions or side chains grafted in the helical regions [22, 50, 51], while our functional motif was embedded in the beta-sheet region. Exciting advances in the area of *de novo* protein design are also yielding many new proteins [11–13], which could then be functionalized with FunFolDes, highlighting the usefulness of this approach. Interestingly, we observed that the functionalized version of TOP7 showed a dramatic decrease in thermodynamic stability as compared to the parent protein. While this observation can be the result of many different factors, it is compelling to interpret it as the “price of function”, meaning that to harbor function, TOP7 was penalized in terms of stability, which would be consistent with our findings in the BINDI benchmark and the experimental studies on stability-function tradeoffs.

Recently, there have also been several *de novo* proteins designed for functional purposes [56]; however, these efforts were limited to linear motifs that carried the functions, and the functionalization was mainly accomplished by side-chain grafting [3, 5], relying on screening a much larger number of designed proteins.

In the light of all the technical improvements, FunFolDes has matured to become a valuable resource for the robust functionalization of proteins using computational design. Here, we presented a number of important findings provided by the detailed benchmarks performed and used the protocol to functionalize proteins in design tasks that are representative of common challenges faced by the broad scientific community when using computational design approaches.

## Materials and methods

### Computational protocol description

Rosetta Functional Folding and Design (FunFolDes) is a general approach for grafting functional motifs into protein scaffolds. It’s main purpose is to provide an accessible tool to tackle specifically those cases in which structural similarity between the functional motif and the insertion region is low, thus expanding the pool of structural templates that can be considered useful scaffolds. This objective is achieved by folding the scaffold after motif insertion while keeping the structural motif static. This process allows the scaffold’s conformation to change and properly adapt to the three-dimensional restrictions enforced by the functional motif. The pipeline of the protocol (summarized in **Fig 1**) proceeds as follows:

#### Selection of the functional motif

A single or multi-segment motif must be selected and provided as an input. In the most common mode of the protocol, dihedral angles, side chain identities and conformations are kept fixed throughout the whole protocol. Conserved sequence length between the motif and the insertion region is not required.

#### Selection of the protein scaffold

Searches for starting protein scaffolds can be achieved, but are not limited to, RMSD similarity matches to the Protein Data Bank (PDB) [57]. The ability of FunFolDes to adapt the scaffold to the needs of the motif widens the structural space of what can be considered a suitable template. Thus, this step requires human intervention and is performed outside of the main protocol.

#### Generation of fragment databases

The usage of fragments lies at the core of many Rosetta protocols, particularly those that perform large explorations of the conformational space required for structure prediction and design. The most standard way of assembling fragment sets is to generate sequence-based fragments using the *FragmentPicker* application [39]. While sequence-based fragments are critical in structure prediction problems, FunFolDes designs have a higher dependency from the structural content of the template rather than its sequence. Thus, we implemented the *StructFragmentMover*, a mover that performs on-the-fly fragment picking based on secondary structure, dihedral angles and solvent accessibility, calculated from the template’s structural information. The typical three- and nine residue-long fragment sets are generated from the global fragment database included in the Rosetta tools release.

#### Generation of constraints

Residue-pair distance and backbone dihedral angle constraints can be extracted from the protein scaffold to guide the folding process. These constraints may include the full-length protein or focus in specific segments while allowing a wider flexibility in other regions. Although not required, the use of constraints greatly increases the quality of the sampling. The protocol can be also made aware of other constraint types (such as cartesian constraints) by properly modifying the score functions applied to the *ab initio* stage [58].

#### Construction of the extended pose

The extended structure is composed of all the segments of the target motif maintaining their native backbone conformation and internal rigid body orientation. The scaffold residues are linearly attached to previously defined insertion points. In multi-segment motif scenarios, the construct will present a chain break between each of the motif-composing segments. This also allows for the segments to be placed into the design in non-consecutive sequence order. Details on how these chain breaks are created can be found in **S1 Text**. Once the extended pose is assembled, it is represented at the centroid level (all side-chain atoms in a single virtual atom) to reduce the computational cost of the simulation.

#### Folding the extended pose

Fragment insertion is performed to accomplish the folding stage. Kinematics of the pose are controlled through the FoldTree [59], a system to control the propagation of the torsion angles applied to a structure. The procedure on how the FoldTree is build and exploited to maintain the appropriate position between different segments of the functional motif is detailed in **S1 Text**. By default, the folding stage is allowed 10 trials to generate a decoy bellow a user defined RMSD threshold. In case the threshold is not reached, this trajectory is skipped and no design will be output.

#### Inclusion of the binding target

If a binding target (protein, nucleic acid or small molecule ligand) is provided, a new FoldTree node is added to the closest residue between the first motif segment and each binding element. Similarly to the multi-segment kinematics, this ensures that the rigid-body orientation between the motif and its target is maintained. FunFolDes can handle simulations with both multi-segment and binding targets simultaneously.

#### Folding post-processing

Folding trajectories are considered successful if they generate structures under a user-defined RMSD threshold of the starting scaffold. In case of a multi-segment motif, a preliminary loop closure will be executed to generate a continuous polypeptide chain, and the kinematic setup maintained to avoid segment displacement during the design step. After the centroid folding stage, the full atom information pose is recovered. All the steps necessary to perform the setup of the extended pose (kinematic setup, folding, post-processing) are carried out by a newly implemented mover called *NubInitioMover*.

#### Protein design and conformational relaxation

The folded structure is subjected to iterative cycles of sequence design [60] and structural relaxation [61] in which the sequence search is coupled with confined conformational sampling [62]. A MoveMap is defined to control backbone dihedrals and side chain conformations of the motif segments and the binding target while allowing for backbone and side-chain sampling of the movable residues (**S1 Text**). TaskOperations are used to avoid undesired mutations in the functional motif.

#### Loop closure

If multi-segment motifs are used, a final loop closure step is required in order to obtain a polypeptide chain without breaks. The *NubInitioLoopClosureMover* performs this last step using the Cyclic Coordinate Descend (CCD) protocol [59], while ensuring that the original conformation and rigid-body orientation of the motifs is maintained. After the closure of each cut-point, a final round of fixed backbone design is performed on the residues of the cut-points and surroundings.

#### Selection, scoring and ranking

Finally, the decoys are ranked and selected according to Rosetta energy, structural metrics (core packing, buried unsatisfied polar atoms, etc) [63], sequence-based predictions such as secondary structure propensity [64] and folding propensity [58] or any other metrics accessible through RosettaScripts (RS). For the *in silico* benchmarks and the design assessments in this work, we used the rstoolbox [65] to produce the statistical analysis and select the best-ranked decoys.

The pipeline components described here represent the most standardized version of the FunFolDes protocol. By means of its integration in RS, different stages can be added, removed or modified to tailor the protocol to the specific needs of the design problem at hand.

### Capturing conformational and sequence changes in small protein domains

To test the ability of FunFolDes to recover the required conformational changes to stabilize a given structural motif, we created a benchmark of 14 target cases of proteins with less than 100 residues, named T01 to T14. Each target case was composed of two structures of the same CATH superfamily [38]. One of the structures was representative of the shared structural features of the CATH family; we called this structure the reference. The second protein within each target case can present two types of structural variations with respect to the reference: I) an insertion or deletion (indel) region and II) a conformational change. Direct structural contacts between these two regions make it so that the indel region is likely the cause for the conformational change. We called this second structure the target (**Fig 2**, **Table 1**).

**Table 1:** Targets included in the conformational and sequence recovery benchmark. For each of the benchmark targets is indicated the CATH superfamily and representatives used in the simulations. (#) indicates the number of segments in the target protein that are considered motif. Motif range indicates the residues considered motif according to the PDB numbering.

ID	CATH	#	reference	target	motif range
**T01**	CATH.3.40.140.10	1	1pgxA	2pw9C	69–73
**T02**	CATH.3.30.310.50	1	3i3wA	4bjuA	464–486
**T03**	CATH.3.30.70.980	1	1lfpA	1mw7A	140–150
**T04**	CATH.3.30.70.100	1	1rjjA	1lq9A	19–45
**T05**	CATH.3.10.20.30	1	2q5wD	2pkoA	49–64
**T06**	CATH.2.30.29.30	1	1c1yB	1h4rA	39–59
**T07**	CATH.3.10.20.90	1	2bkfA	2al6B	115–119
**T08**	CATH.3.10.20.90	1	1wj4a	1wiaA	181–200
**T09**	CATH.3.10.20.90	1	3ny5B	3phxB	100–121
**T10**	CATH.3.10.20.310	1	2x8xX	2qdfA	103–121
**T11**	CATH.3.10.320.10	1	4p5mA	2bc4C	56–66
**T12**	CATH.2.40.40.20	1	1cr5B	2pjhB	119–142
**T13**	CATH.2.40.40.20	2	1cr5B	2pjhB	119–142, 168–173
**T14**	CATH.3.30.110.40	1	1jdqA	3lvjC	14–37

For each template protein we generated approximately 10000 decoys with FunFolDes by folding the target with the following conditions: 1) the indel region was considered as the motif, meaning that its structural conformation was kept fixed and no mutations allowed; 2) residue-pair distance constraints were derived from the secondary structure elements conserved between reference and the target (constrained region); 3) the region of the protein which showed the largest structural variations (query region) was constraint-free throughout the simulation.

FunFolDes simulations were compared with constrained *ab initio (*cst*-ab initio)* simulations, the key difference being that the cst-*ab initio* simulations allowed for backbone flexibility in the motif region. The comparison between both approaches provides insights on the effects of a static segment in the folding trajectory of the polypeptide chain. In both scenarios a threshold was set after the folding stage where only decoys that had less than 5 Å RMSD from the template were carried to the design stage.

The importance of the input fragments was assessed in our benchmark. Both protocols were tested with sequence-based fragments from *FragmentPicker* and structure-based fragments generated on-the-fly by FunFolDes. Comparison between the two types of fragments provides insight into how to utilize FunFolDes in the most productive manner.

Structural recovery was evaluated by RMSD with the target structure. Global RMSD, understood as the minimum possible RMSD given the most optimal structural alignment, was used to assess the overall structural recovery of each decoy population. Local RMSD, was evaluated for the unconstrained (query) region and the motif by aligning each decoy to the template through the constrained segments (excluding the motif). This metric aimed to capture the specific conformational changes required to accommodate the motif into the structure (**Fig 2B**, **S1B Fig**).

Sequence recovery was evaluated through two different criteria, sequence associated statistics and Hidden Markov Model (HMM) [41]. For the sequence associated statistics, we quantified sequence identity and similarity according to BLOSUM62 for the core residues of each protein, as defined by Rosetta’s *LayerSelector* [7]. Motif residues, that were not allowed to mutate, were excluded from the statistics. In the second criteria, position specific scoring matrices with inter-position dependency known as Hidden Markov Model (HMM) were used to evaluate fold specific sequence signatures. In this case, the closest HMM to the template structure provided by CATH was used to query the decoys and identify those that matched the HMM under two conditions: I) an e-value under 10 and II) a sequence coverage over 50%. Although these conditions are wide, they were within the ranges found between members of CATH superfamilies with high structural and sequence variability like the ones used in the benchmark.

### Target-biased design of protein binders

To assess the performance of FunFolDes in the presence of a binding target we recreated the design of BINDI as a binder for BHRF1 [21], the BHRF1 binding motif from the BIM-BH3 protein (PDB ID:2WH6 [66]) was inserted into a previously described 3-helix bundle scaffold (PDB ID:3LHP [3]).

Four different design simulations were performed, one without the binder (*no_target*) and three in the presence of the binder (*static*, *pack* and *packmin*). The difference between the last three relates to how the binding target was handled. In the *static* simulations the binding target was kept fixed and no conformational movement in the side chains was allowed throughout the protocol. In the *pack* simulations the side chains of the binding target were repacked during the binder design stage. Finally, in the *packmin* simulations the binding target side-chains were allowed to repack and both side-chains and backbone were subjected to minimization. In all cases, the two terminal residues on each termini of the binding motif were allowed backbone movement to optimize the insertion in the 3-helix bundle scaffold. For each of these simulations, approximately 20000 decoys were generated.

For the *no_target* simulations the FunFolDes designs were docked to BHRF1 using the inserted motif as guide to assess their complementarity and interface metrics. In all the simulations, a final round of global minimization was performed, where both proteins of the complex were allowed backbone flexibility. During this minimization, the rigid-body orientation between the design and target was kept fixed. The final ΔΔG of the complexes was measured after the minimization step to enable comparisons between the *no_target* decoys and the remaining simulation modes. Structural changes related to this minimization step were evaluated as the global RMSD between each structure before and after the process, this measure is referred to as RMSD drift.

Structural evaluation includes global RMSD against the BINDI crystal structure (PDB ID: 4OYD [21]) as well as local RMSDs against regions of interest in BINDI. In the Local-RMSD calculations the structures were aligned through the inserted motif, as its conformation and orientation relative to BINDI were kept fixed throughout all simulations. The local RMSD analysis was performed over all the helical segments contained in the structures (all H), which provided a measurement of the structural shifts on the secondary structure regions of the designs.

To evaluate the sequence recovery we leveraged BINDI’s saturation mutagenesis data analyzed by deep sequencing performed by Procko *et al* [21]. The experimental fitness of each mutation was summarized in a score matrix where a score was assigned to each amino-acid substitution for the 116 positions of the protein (**S2C Fig**). In summary, point mutations that improved BINDI’s binding to BHRF1 are assigned positive scores while deleterious mutations present negative values. These scores were computed based on experimental data where the relative populations of each mutant were compared between a positive population of cells displaying the designs (binders) and negative populations (mutants that display but don’t bind), these experiments have been described in detail elsewhere [21]. Upon normalization by the BINDI sequence score, a position sequence specific matrix (PSSM) was created. Like the original data, this matrix also assigns a positive score to each point mutation if it resulted in an improved binding for the design. This normalization provides a score of 0 for the BINDI sequence, which is useful as a reference score.

### Repurposing naturally occurring folds for a new functions

To experimentally validate the capabilities of FunFolDes and insert functional sites in structurally distant templates, we grafted the 11 residues from the site II epitope from the Respiratory Syncytial Virus (RSV) protein F (PDB ID:3IXT [46]), residues 256 to 276 in chain P (NSELLSLINDMPITNDQKKLMSN), into heterologous scaffolds. This is a continuous, single segment, helix-loop-helix conformation epitope. The main objective was to challenge the capabilities of FunFolDes to reshape the structure of the scaffold to the requirements of the functional motif. We searched for insertion segments with RMSDs towards the site II structure higher than 2 Å.

The structural searches were performed using MASTER [47] where we used the full-length site II segment as a query against a subset of 17539 protein structures from the PDB, composed of 30% non-redundant sequences included in the MASTER distribution. The RMSD between the query and segments on the scaffolds were assessed using backbone C_α_s. All matches with RMSD_Cα_ < 5.5 Å relative to site II were further filtered by protein size, where only proteins between 50 and 100 residues were kept. These scaffolds were then ranked regarding antibody-binding compatibility, where each match was realigned to the antibody–epitope complex and steric clashes between all glycine versions of the scaffold and antibody were quantified using Rosetta. All matching scaffolds with ΔΔG values above 100 REU were discarded under the assumption that their compatibility with the antibody binding mode was too low. The remaining scaffolds were visually inspected and PDB ID: 1kx8 [48] (RMSD_Cα_ = 2.37 Å) was selected for design with FunFolDes. The twenty-one residues from the site II epitope (motif) as present in 3IXT were grafted into a same sized segment (residues 79–100) of 1kx8 using the *NubInitioMover*. Up to three residues in each insertion region of the motif were allowed backbone flexibility in order to model proper conformational transitions in the insertion points. Atom pair constraints with a standard deviation of 3 Å were defined for all template residues, leaving the motif segment free of constraints. The generous standard deviation was defined to allow for necessary conformational changes to retrofit the motif within the topology. The total allowed deviation from the template was limited to 5 Å to ensure the retrieval of the same topology. In this design series we used sequence-based fragments generated with the 1kx8 native sequence. Three cycles of design/relax were performed on the template residues with the *FastDesignMover*.

A first generation of 12500 designs was ranked according to Rosetta energy. From the top 50 decoys, only one presented the motif without distortions on the edges derived from the allowed terminal flexibility. This decoy was used as template on the second generation of FunFolDes to enhance the sampling of properly folded conformations, with the same input conditions as before.

In the second generation, the top 50 decoys according to Rosetta energy were further optimized through additional cycles of design/relax. The final designs were again selected using a composite filter based on Rosetta energy (top 50), buried unsatisfied polar atoms (<15), cavity volume (< 75 Å^3^) and we obtained a final set of 15 candidates from which we prioritized 6 upon the inspection of the computational models. In addition, we also quantified the secondary structure prediction using PSIPRED [64], all the tested designs had more 65% (ranging from 65% to 92%) of the residues with correct secondary structure prediction. The final designs were manually optimized, this process entailed the removal of designed hydrophobic residues in solvent exposed positions, in this designs series we performed between 2 and 4 mutations obtaining 7 designs from the previous 6. After the initial characterization, designs with added disulfide bridges were generated to improve protein stability and affinity (**S3 Fig**, **S4 Fig**). To do so, we use the Rosetta *DisulfidizeMover*, which screened the designed models for pairs of residues with favourable three-dimensional orientations to host disulfide bonds. Upon the placement of the disulfide bond, the neighbouring residues within 10 Å of the disulfide, were designed to optimize the residue interactions and improve the packing of the designed region.

### Functionalization of a functionless fold

In a second effort to test the design capabilities of FunFolDes we sought to insert a functional motif in one of the first de novo designed proteins–TOP7 (PDB ID: 1QYS [13])

Six residues from the complex between the antibody 101F and the peptide-epitope, corresponding to residues 429–434 in chain P (RGIIKT) on the full-length RSV F protein [49], were grafted into the edge strand of the TOP7 backbone using FunFolDes. The choice between epitope and hosting scaffold was made based on the secondary structure adopted by the epitope and the structural compatibility of TOP7, the RMSD_Cα_ between the epitope an the insertion segment was 2.07 Å.

To ensure that the majority of the β-strand secondary structure was maintained throughout the grafting protocol, the epitope motif was extended by one residue and a designed 4-residue β-strand (KVTV) pairing with the backbone of the C-terminal epitope residues was co-grafted as a discontinuous segment into the adjacent strand of the TOP7 backbone. With this strategy we circumvented a Rosetta sampling limitation, where often times extensive sets of constraints to achieve backbone hydrogen-bonds on beta-strands are necessary [8]. After defining the motif consisting of the epitope plus the pairing strand and the sites of insertion on the TOP7 scaffold, FunFolDes was used to graft the motif.

Backbone flexibility was allowed for the terminal residues of the functional motif and a β-turn connection between the two strands was modelled during the folding process (*NubInitioMover*). During the folding process, the 101F antibody was added to the simulation in order to limit the explored conformational space productive for binding. Finally, the *NubInitioLoopClosureMover* was applied to ensure that a proper polypeptide chain was modelled and no chain-breaks remained, a total of 800 centroid models were generated after this stage. Next, we applied an RMSD filter to select scaffolds with similar topology to TOP7 (< 1.5 Å) and a hydrogen bond long-range backbone score (HB_LR term) to favour the selection of proteins with proper beta-sheet pairing. The top 100 models according the HB_LR score and <1.5 Å to TOP7, were then subjected to an iterative sequence-design relax protocol, alternating fixed backbone side-chain design and backbone relaxation using the *FastDesignMover*. Two different design strategies were pursued: I) partial design—amino acid identities of the C-terminal half of the protein (residues 45 through 92) were retained from TOP7 while allowing repacking of the side chains and backbone relaxation; II) full-design—the full sequence space in all residues of the structure (with the exception of the 101F epitope) was explored. No backbone or side chain movements were allowed in the 6-residue epitope segment whereas the adjacently paired β-strand was allowed to both mutate and relax. Tight Cα atom-pair distance constraints (standard deviation of 0.5 Å) were used to restrain movements of the entire sheet throughout the structural relaxation iterations.

From the 100 designs generated, only those that passed a structural filter requiring 80% beta-sheet secondary structure composition after backbone relaxation were selected for further analysis.

The 93 designs passing this filter were evaluated with a composite filter based on REU score (Top 50), hydrogen-bond long-range backbone interactions (< -113) and core packing (> 0.7). The selected designs were finally submitted to human-guided optimisation to correct for hydrophobic residues that were designed in solvent exposed positions (1–3) and shortening of the connecting loop between the two inserted strands using the Rosetta Remodel application [67].

Interestingly, in an attempt to reproduce the same grafting exercise with *MotifGraftMover* [55], this resulted in non-resolvable chain breaks when trying to graft either the two segment-motif or the epitope alone into the TOP7 scaffold.

### Protein expression and purification

DNA sequences of the designs were purchased from Twist Bioscience. For bacterial expression the DNA fragments were cloned via Gibson cloning into a pET21b vector containing a C-terminal His-tag and transformed into *E*. *coli* BL21(DE3). Expression was conducted in Terrific Broth supplemented with ampicillin (100 μg/ml). Cultures were inoculated at an OD_600_ of 0.1 from an overnight culture and incubated at 37°C with a shaking speed of 220 rpm. After reaching OD_600_ of 0.7, expression was induced by the addition of 1 mM IPTG and cells were further incubated for 4-5h at 37°C. Cells were harvested by centrifugation and pellets were resuspended in lysis buffer (50 mM TRIS, pH 7.5, 500 mM NaCl, 5% Glycerol, 1 mg/ml lysozyme, 1 mM PMSF, 1 μg/ml DNase). Resuspended cells were sonicated and clarified by centrifugation. Ni-NTA purification of sterile-filtered (0.22 μm) supernatant was performed using a 1 ml His-Trap FF column on an ÄKTA pure system (GE healthcare). Bound proteins were eluted using an imidazole concentration of 300 mM. Concentrated proteins were further purified by size exclusion chromatography on a Superdex 75 300/10 GL or a Hiload 16/600 Superdex 75 pg column (GE Healthcare) using PBS buffer (pH 7.4) as mobile phase.

For IgG expression, heavy and light chain DNA sequences were cloned separately into pFUSE-CHIg-hG1 (InvivoGen) mammalian expression vectors. Expression plasmids were co-transfected into HEK293-F cells in FreeStyle medium (Gibco) using polyethylenimine (Polysciences) transfection. Supernatants were harvested after 1 week by centrifugation and purified using a 5 ml HiTrap Protein A HP column (GE Healthcare). Elution of bound proteins was accomplished using a 0.1 M glycine buffer (pH 2.7) and eluents were immediately neutralized by the addition of 1 M TRIS ethylamine (pH 9). The eluted IgGs were further purified by size exclusion chromatography on a Superdex 200 10/300 GL column (GE Healthcare) in PBS buffer (pH 7.4). Protein concentrations were determined by measuring the absorbance at 280 nm using the sequence calculated extinction coefficient on a Nanodrop (Thermo Scientific).

### Circular Dichroism (CD)

Far-UV circular dichroism spectra of designed scaffolds were collected between a wavelength of 190 nm to 250 nm on a Jasco J-815 CD spectrometer in a 1 mm path-length quartz cuvette. Proteins were dissolved in PBS buffer (pH 7.4) at concentrations between 20 μM and 40 μM. Wavelength spectra were averaged from two scans with a scanning speed of 20 nm min^-1^ and a response time of 0.125 sec. The thermal denaturation curves were collected by measuring the change in ellipticity at 220 nm from 20 to 95°C with 2 or 5°C increments.

### Size-exclusion Chromatography combined with Multi-Angle Light-Scattering (SEC-MALS)

Multi-angle light scattering was used to assess the monodispersity and molecular weight of the proteins. Samples containing between 50–100 μg of protein in PBS buffer (pH 7.4) were injected into a Superdex 75 300/10 GL column (GE Healthcare) using an HPLC system (Ultimate 3000, Thermo Scientific) at a flow rate of 0.5 ml min^-1^ coupled in-line to a multi-angle light scattering device (miniDAWN TREOS, Wyatt). Static light-scattering signal was recorded from three different scattering angles. The scatter data were analyzed by ASTRA software (version 6.1, Wyatt)

### Surface Plasmon Resonance (SPR)

To determine the dissociation constants of the designs to the mota or 101F antibodies, surface plasmon resonance was used. Experiments were performed on a Biacore 8K at room temperature with HBS-EP+ running buffer (10 mM HEPES pH 7.4, 150 mM NaCl, 3mM EDTA, 0.005% v/v Surfactant P20) (GE Healthcare). Approximately 1200 response units of mota or 101F antibody were immobilized via amine coupling on the methyl-carboxyl dextran surface of a CM5 chip (GE Healthcare). Varying protein concentrations were injected over the surface at a flow rate of 30 μl/min with a contact time of 120 sec and a following dissociation period of 400 sec. Following each injection cycle, ligand regeneration was performed using 3 M MgCl_2_ (GE Healthcare). Data analysis was performed using 1:1 Langmuir binding kinetic fits within the Biacore evaluation software (GE Healthcare).

## Availability

FunFolDes is available as part of the Rosetta software suite and is fully documented in the Rosetta Commons documentation website as one of the Composite Protocols. All data and scripts necessary to recreate the analysis and design simulations described in this work are available at https://github.com/lpdi-epfl/FunFolDesData.

## Supporting information

S1 Text FoldTree and MoveMap. Description of the specific setup of the FoldTree and MoveMap in order to properly guide the folding process in FunFolDes.
(PDF)

S1 Fig Structural composition and overall results of the benchmark targets. A) Percentage of secondary structure type, motif and query region in the overall structures. B) Full structure RMSD (Overall RMSD) and local RMSD for the query region (Query Region) between the decoy populations and their respective targets. FunFolDes tends to outperform cst–*ab initio* in all scenarios and the structure-based fragments yield decoy population with lower mean RMSDs, albeit with small differences relative to the sequence-based fragments.
(TIF)

S2 Fig Target-biased folding and design: Structural features of the modeled designs and saturation mutagenesis data used for sequence recovery benchmark. A) Quantification of the percentage of decoys compatible with a design-target binding conformation for the different simulation modes. The simulations performed without the target yield a very low percentage of binding compatible conformations. After minimization, this percentage increases with significant structural drifts. B) The initial template is a 3-helix bundle structure, the slight shift needed to adopt a binding-compatible conformation produces only a small global RMSD. C) Graphical representation of the deep-sequencing data as a position-specific score matrix. Black borders highlight the native BINDI residue type for each position. Mutations for which no data were obtained, likely reflect that these protein variants were unable to fold or display at the surface of yeast and were assigned the lowest score of -5.
(TIF)

S3 Fig Structural and sequence evaluation of the computational designs. Assessment of structural and sequence features: Rosetta Energy, packing score (packstat) [63], cavity volume, Buried UNSatisfied polar atoms and secondary structure prediction (PSIPRED) for the template and the computational designs. Each native template (green diamond and vertical dashed line) and design (yellow and blue circles) are compared against a set of non-redundant minimized structures of similar size (± 15 residues). A) Due to its natural function, 1kx8 presents of a large cavity to bind its hydrophobic ligands. As such, the structure presents generally low scores as compared to computationally designed proteins. B) Distributions of the structural and sequence features of natural proteins and the TOP7 series of designs.
(TIF)

S4 Fig Examples of experimental characterization performed for other variants on the 1kx8 design series. A) CD wavelength spectra (left column), thermal denaturations (middle column) and SPR binding assays with the mota antibody (right column) were performed. B) Global sequence alignment of the wild-type protein 1kx8 and the computationally designed sequences. Red positions highlight the site II epitope insertion. Green positions highlight the cysteines performing the disulfide bridges. The two positions that consistently kept the original residue type of 1kx8 are highlighted in bold.
(TIF)

S5 Fig In silico assessment of 1kx8_d2 and TOP7_full computational designs. A) *Ab initio* folding simulations for wild-type 1kx8 (left) and design 1kx8_d2 (center), ensembles generated by relaxing the starting structures are shown in orange. The inability of 1kx8 to form a proper folding funnel could be explained by the big internal cavity of the protein due to its fatty-acid binding pocket. Docking-minimization simulations (right) performed with the top50 scoring *ab initio* decoys. The docking simulations reveal a similar binding configuration between the peptide motif and the full design, and ΔΔGs are within a similar range to those of the native peptide antibody complex. B) Same simulations as described in A) for wild-type TOP7 (left) and TOP7_full (center). We observe energetically favorable folding funnels for both wildtype and design. The docking simulations showed that the complex between the design and the antibody is formed in a similar structural configuration to the peptide-antibody complex achieving a similar range of ΔΔGs.
(TIF)

S6 Fig Experimental characterization of TOP7_variants. A) Experimental characterization for the TOP7_partial design: SEC-MALS elution profile (left column); CD wavelength scan spectrum; SPR binding assays with the 101F antibody (right column). The TOP7_partial CD spectrum is notably different from WT TOP7 and the TOP7_full design. B) Global sequence alignment of the wild-type protein TOP7 and the computationally designed sequences. Red positions highlight the site IV epitope insertion.
(TIF)
