# Supplementary material for: Rosetta FunFolDes – A general framework for the computational design of functional proteins
Source: PLoS Comput Biol. 2018 Nov 19;14(11):e1006623. doi: 10.1371/journal.pcbi.1006623 (PMC6277116; doi:10.1371/journal.pcbi.1006623)
Supplement: S1 Text — Description of the specific setup of the FoldTree and MoveMap in order to properly guide the folding process in FunFolDes. (PDF) [file pcbi.1006623.s001.pdf]

Rosetta FunFolDes – a general framework for the computational design of functional proteins

Jaume Bonet¶, Sarah Wehrle¶, Karen Schriever¶, Che Yang¶, Anne Billet, Fabian Sesterhenn, Andreas Scheck, Freyr Sverrisson, Barbora Veselkova, Sabrina Vollers, Roxanne Lourman, Mélanie Villard, Stéphane Rosset, Thomas Krey, Bruno E. Correia.

S1 TEXT: FOLDTREE AND MOVEMAP **2**

FoldTree: Basics 2
MoveMap: Basics 3
FunFolDes: Single-segment motif 4
FunFolDes: Multi-segment motif 4
FunFolDes: Target binder 5

REFERENCES **7**

Supplementary Figure A. Default FoldTree. 2
Supplementary Figure B. FoldTree for two bound proteins. 2
Supplementary Figure C. Single-segment motif FunFolDes design. 4
Supplementary Figure D. Multi-segment motif FunFolDes design. 5
Supplementary Figure E. Single-segment motif design with binder. 6
Supplementary Figure F. Multi-segment motif design with binder. 6

S1 Text: FoldTree and MoveMap

FoldTree: Basics

In Rosetta, the FoldTree [1] is a graph representation of the connectivity of a structure that controls its kinematics through the propagation of changes to the torsion angles applied to the structure. An extensive explanation of the FoldTree can be found in the official Rosetta documentation:

https://www.rosettacommons.org/demos/latest/tutorials/fold_tree/fold_tree.

For the purposes of this work, the following properties are important to highlight:

1. A FoldTree has to cover the totality of the structure. This includes all protein chains and, if applicable, small-molecules.
2. FoldTrees are not cyclic and start at a given node dubbed as **root**.
3. A FoldTree contains two main types of connectivity: **peptide edges** (indicated with the value -1), that represent peptide connections through which the angle torsions are propagated, and **jumps** (indicated with consecutive positive values), that represent non polymeric connections between different points of the fold tree and that translate in the 3D space to the maintenance of defined rigid body orientations between the segments they connect.
4. The ends on a continuous stretch of peptide edges are labeled as **cutpoints**. These could represent either **chain ends** (like N- and C-terminal) or **chain breaks**, discontinuities inside a single chain that avoid the transfer of torsion changes.

Thus, a typical FoldTree for a 100-residue protein would by default look like this:

```
FOLD_TREE  EDGE 1 100 -1
```

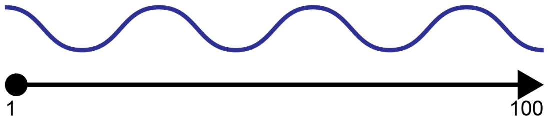

Supplementary Figure A. Default FoldTree. *By default a FoldTree (black) will transfer the torsion effects from the first to the last residue of a given protein structure (blue).*

The FoldTree for two bound 100-residue proteins would be as follows:

```
FOLD_TREE  EDGE 1 100 -1  EDGE 100 101 1  EDGE 101 200 -1
```

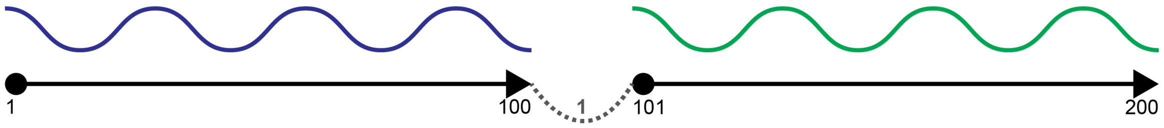

Supplementary Figure B. FoldTree for two bound proteins. *When two proteins (blue and green) form a complex, a jump between the two chains is defined, maintaining the rigid body orientation between the two proteina and creating a FoldTree to describe the full complex.*

MoveMap: Basics

The MoveMap [1] definition controls the degrees of freedom of each individual residue. The degrees of freedom can be controlled at two levels backbone (**BB**: True/False), that defines whether or not backbone angles (ϕ, ψ) can be altered; side-chain (**CHI**: True/False) that defines the ability of the side chain angles (χ_n) to be changed.

Thus, a flexible backbone relaxation with full mobility on a 100-residue protein will be defined as:

```
RESIDUE 1 100 BBCHI
```

While a fixed backbone with only side-chain repacking would be described as:

```
RESIDUE 1 100 CHI
```

In combination with the FoldTree, there is a third variant definition in the MoveMap: the **JUMP** (True/False), defines the ability for the residues at both sides of a FoldTree jump to change their relative positions.

For example, given our previous two-protein FoldTree:

```
FOLD_TREE  EDGE 1 100 -1  EDGE 100 101 1  EDGE 101 200 -1
```

Applying structure relaxation with a MoveMap such as

```
RESIDUE 1 100 BBCHI
RESIDUE 101 200 BBCHI
JUMP 1 YES
```

will allow for the proteins to change their rigid body orientation to each other, while

```
RESIDUE 1 100 BBCHI
RESIDUE 101 200 BBCHI
JUMP 1 NO
```

will keep that orientation fixed.

FunFolDes: Single-segment motif

The most straightforward application of FunFolDes is the grafting of a single-segment motif.

Given that we want to insert an 11-residue motif between positions 25 to 35 in a 100-residue protein.

The final FoldTree will look like this:

```
FOLD_TREE  EDGE 30 1 -1  EDGE 30 100 -1
```

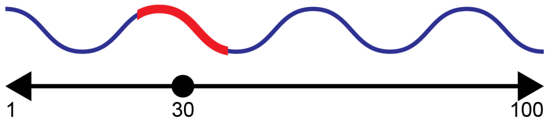

Supplementary Figure C. Single-segment motif FunFolDes design. *The root of the FoldTree is placed in the middle of the motif (red) and expands towards both ends of the protein.*

While the default MoveMap will be:

```
RESIDUE 1 24 BBCHI
RESIDUE 25 35 NO
RESIDUE 36 100 BBCHI
```

In this case, the root of the FoldTree is placed in the middle of the grafted segment and expands towards both N- and C-terminus of the protein. Combined with the MoveMap restrictions, this setup allows for the segments at each side of the motif to refold while the motif stays static.

FunFolDes: Multi-segment motif

A second level of complexity is the grafting of multi-segment functional motifs, i.e., of non-contiguous structural fragments.

In this scenario, a continuous tree in which torsion changes are propagated in the region between the two insertions would result in changes regarding the rigid body orientation between the individual structural motifs, thus interfering in the proper mimicry of the full functional motif. Therefore, a **cutpoint** has to be introduced in this segment to avoid this propagation of torsional changes. As this has to happen between each pair of inserted segments, the number of chain breaks in the FoldTree is:

$$count_{chainbreaks} = count_{segments} - 1 \quad \text{Eq1}$$

To select the appropriate region where to generate the cutpoint, FunFolDes uses the secondary structure assignment provided by Rosetta's internal implementation of DSSP [2] to locate a loop region between the two motif segments. If found, the midpoint of the loop is selected as the cutpoint. When no loop is found, the cutpoint is set in the middle residue between the two segments.

Once the cutpoints have been chosen, a FoldTree root is placed in the middle of each grafted motif segment and expanded in both directions towards their flanking cutpoints (here N- and C-terminus are considered as cutpoints, although they are not chain breaks). Finally, jumps span from the root of the first segment (in sequential order) towards the rest of the roots of the other segments, creating a fully connected FoldTree.

As an example, assuming that we want to graft two segments into a 100-residue protein, one between residues 25 and 35 and another between residues 60 and 70. Following the previous explanation, and premising a loop region in residues 45-50, the FoldTree is defined as:

```
FOLD_TREE  EDGE 30 1 -1  EDGE 30 47 -1  EDGE 30 65 1  EDGE 65 48 -1  EDGE 65 100 -1
```

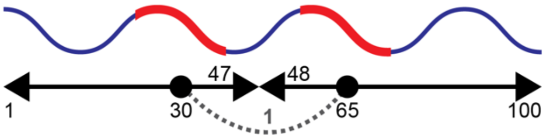

Supplementary Figure D. Multi-segment motif FunFolDes design. *Two bi-directional FoldTrees are rooted in their dependent segment moti (red)f. The roots are joined by a jump in order to form a complete FoldTree and maintain the rigid body orientation of the motif segments .*

In order to keep different segments in a FunFolDes run fixed within themselves and with respect to each other, the MoveMap needs to be set so that the internal conformation of the motif and the jumps cannot be altered:

```
RESIDUE 1 24 BBCHI
RESIDUE 25 35 NO
RESIDUE 36 59 BBCHI
RESIDUE 60 70 NO
RESIDUE 71 100 BBCHI
JUMP 1 NO
```

<u>FunFolDes: Target binder</u>

First of all, it is important to highlight that a target binder (if available) has to be provided through the same PDB file [3] as the functional motif, to maintain the exact rigid body orientation between the motif and the target binder.

From the point of view of the FoldTree, adding one or multiple binders (e.g. the two protein chains of a target antibody) is easier than inserting multiple segments. Simply put, for each binder chain, the closest residue to the root of the FoldTree (located in the first segment of the motif) is identified and set up as root of an individual FoldTree that spans in both directions of the binder chain. Finally, a jump is set up between the old and the new root to unify the two FoldTrees into one.

Thus, if we add a 100-residue partner to our single-segment motif design (assuming the closest residue to the motif is residue 50 of the binding partner), the FoldTree will transform from

```
FOLD_TREE  EDGE 30 1 -1  EDGE 30 100 -1
```

to

```
FOLD_TREE  EDGE 30 1 -1  EDGE 30 100 -1  EDGE 30 150 1  EDGE 150 101 -1  EDGE 150 200 -1
```

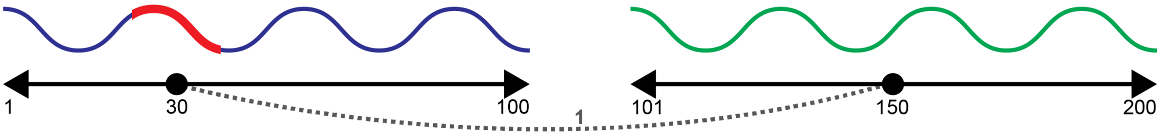

Supplementary Figure E. Single-segment motif design with binder. *The two proteins are attached through a jump defined between the tree's root in the motif and the closest residue (in space) from the target binder.*

Repeating the same exercise on our two-segment motif design will change the original FoldTree

```
FOLD_TREE  EDGE 30 1 -1  EDGE 30 47 -1  EDGE 30 65 1  EDGE 65 48 -1  EDGE 65 100 -1
```

to

```
FOLD_TREE  EDGE 30 1 -1  EDGE 30 47 -1  EDGE 30 65 1  EDGE 65 48 -1  EDGE 65 100 -1
           EDGE 30 150 2  EDGE 150 101 -1  EDGE 150 200 -1
```

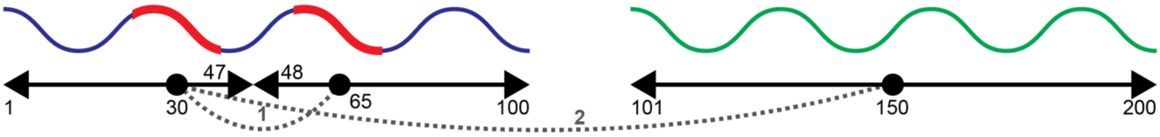

Supplementary Figure F. Multi-segment motif design with binder. *The FoldTree construction follows the same logic as before, adding one more jump to keep the target binder in place with respect to the functional motif.*

As before, setting the jumps of the MoveMap to static is key to ensure that motif and target binder are correctly positioned with respect to each other. Furthermore, it is necessary to prevent the target binder(s) from moving at all during this process. Because of that, the MoveMap will look like

```
RESIDUE 1 24 BBCHI
RESIDUE 25 35 NO
RESIDUE 36 100 BBCHI
RESIDUE 101 200 NO
JUMP 1 NO
```

for the single-segment motif with binder and

```
RESIDUE 1 24 BBCHI
RESIDUE 25 35 NO
RESIDUE 36 59 BBCHI
RESIDUE 60 70 NO
RESIDUE 71 100 BBCHI
RESIDUE 101 200 NO
JUMP 1 NO
JUMP 2 NO
```

for the multi-segment motif with binder.

References

1. Wang C, Bradley P, Baker D. Protein-protein docking with backbone flexibility. J Mol Biol. 2007;373(2):503-19. Epub 2007/09/11. doi: 10.1016/j.jmb.2007.07.050. PubMed PMID: 17825317.
2. Kabsch W, Sander C. Dictionary of protein secondary structure: pattern recognition of hydrogen-bonded and geometrical features. Biopolymers. 1983;22(12):2577-637. Epub 1983/12/01. doi: 10.1002/bip.360221211. PubMed PMID: 6667333.
3. Rose PW, Prlic A, Altunkaya A, Bi C, Bradley AR, Christie CH, et al. The RCSB protein data bank: integrative view of protein, gene and 3D structural information. Nucleic Acids Res. 2017;45(D1):D271-D81. Epub 2016/10/30. doi: 10.1093/nar/gkw1000. PubMed PMID: 27794042; PubMed Central PMCID: PMCPMC5210513.
