# Supplementary figures and images for: Rosetta FunFolDes – A general framework for the computational design of functional proteins
Source: PLoS Comput Biol. 2018 Nov 19;14(11):e1006623. doi: 10.1371/journal.pcbi.1006623 (PMC6277116; doi:10.1371/journal.pcbi.1006623)

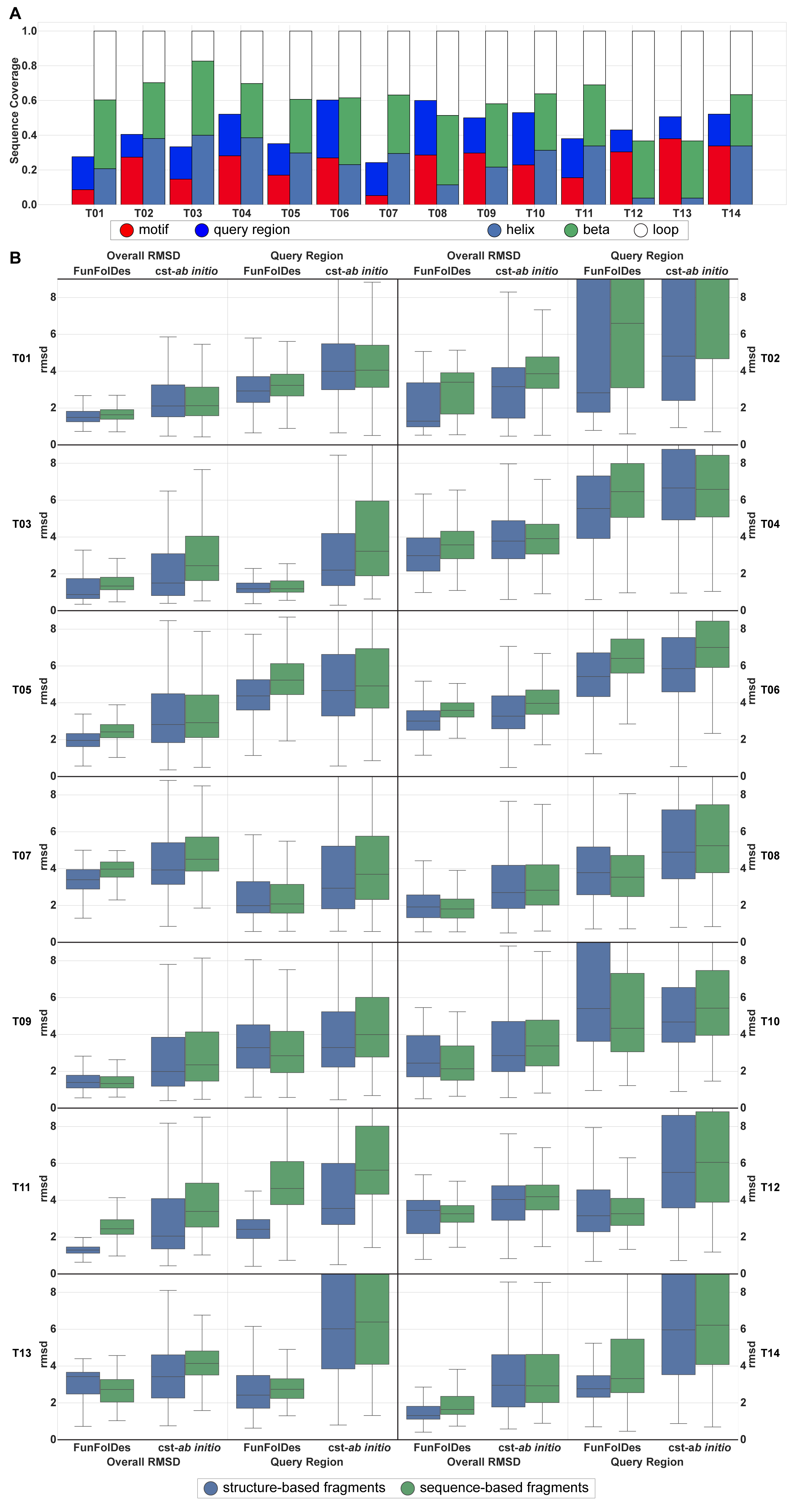

Supplement: S1 Fig — A) Percentage of secondary structure type, motif and query region in the overall structures. B) Full structure RMSD (Overall RMSD) and local RMSD for the query region (Query Region) between the decoy populations and their respective targets. FunFolDes tends to outperform cst–ab initio in all scenarios and the structure-based fragments yield decoy population with lower mean RMSDs, albeit with small differences relative to the sequence-based fragments. (TIF) [file pcbi.1006623.s002.tif]

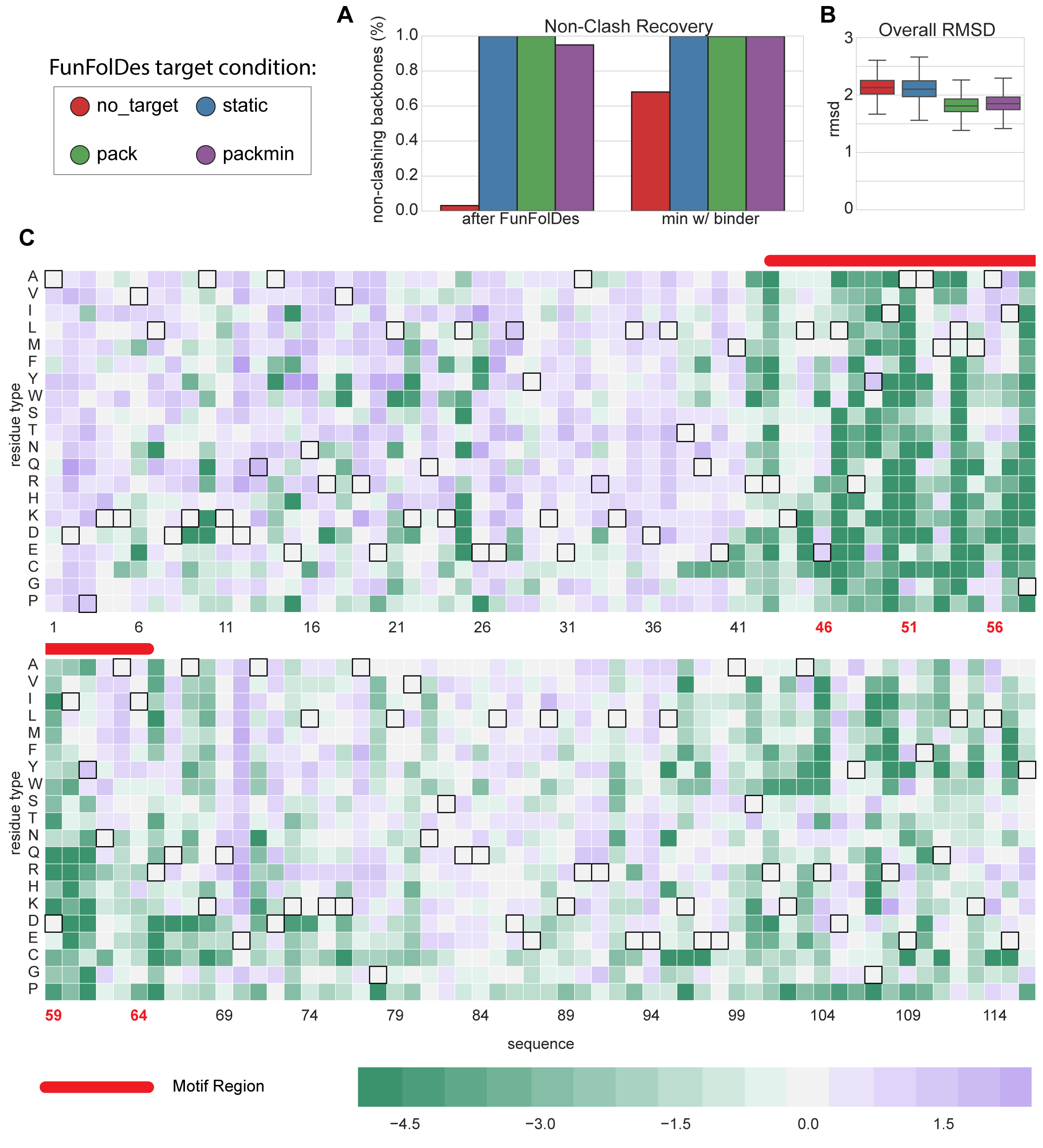

Supplement: S2 Fig — A) Quantification of the percentage of decoys compatible with a design-target binding conformation for the different simulation modes. The simulations performed without the target yield a very low percentage of binding compatible conformations. After minimization, this percentage increases with significant structural drifts. B) The initial template is a 3-helix bundle structure, the slight shift needed to adopt a binding-compatible conformation produces only a small global RMSD. C) Graphical representation of the deep-sequencing data as a position-specific score matrix. Black borders highlight the native BINDI residue type for each position. Mutations for which no data were obtained, likely reflect that these protein variants were unable to fold or display at the surface of yeast and were assigned the lowest score of -5. (TIF) [file pcbi.1006623.s003.tif]

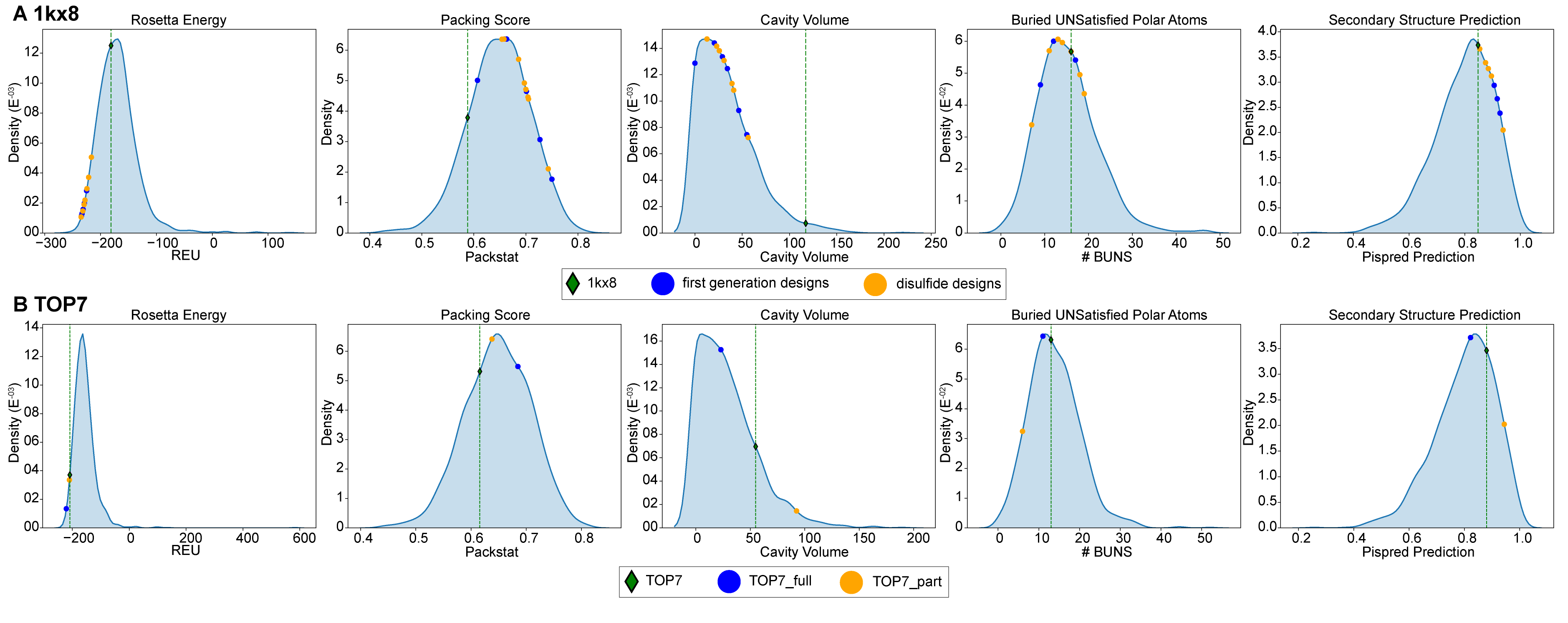

Supplement: S3 Fig — Assessment of structural and sequence features: Rosetta Energy, packing score (packstat) [63], cavity volume, Buried UNSatisfied polar atoms and secondary structure prediction (PSIPRED) for the template and the computational designs. Each native template (green diamond and vertical dashed line) and design (yellow and blue circles) are compared against a set of non-redundant minimized structures of similar size (± 15 residues). A) Due to its natural function, 1kx8 presents of a large cavity to bind its hydrophobic ligands. As such, the structure presents generally low scores as compared to computationally designed proteins. B) Distributions of the structural and sequence features of natural proteins and the TOP7 series of designs. (TIF) [file pcbi.1006623.s004.tif]

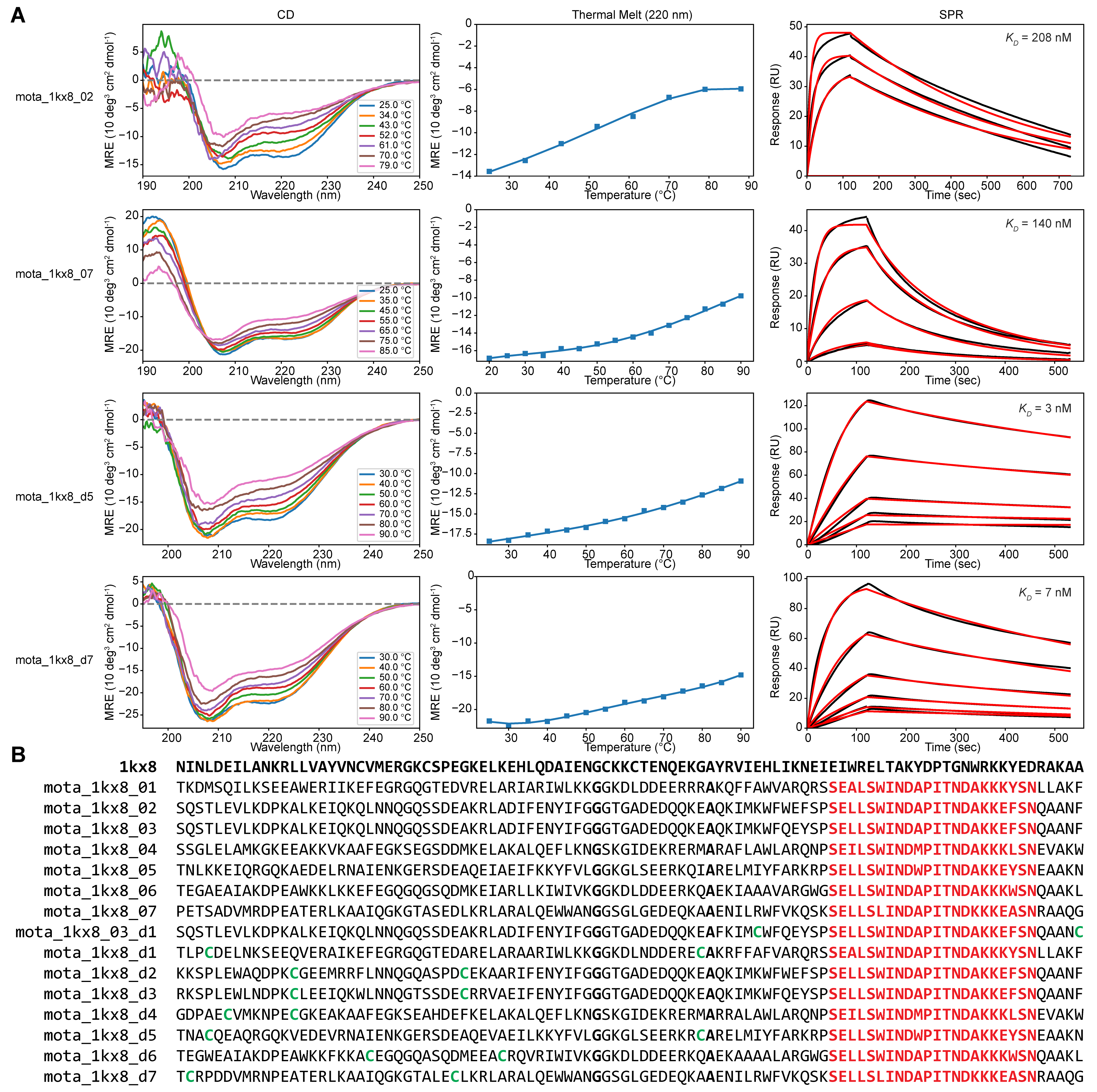

Supplement: S4 Fig — A) CD wavelength spectra (left column), thermal denaturations (middle column) and SPR binding assays with the mota antibody (right column) were performed. B) Global sequence alignment of the wild-type protein 1kx8 and the computationally designed sequences. Red positions highlight the site II epitope insertion. Green positions highlight the cysteines performing the disulfide bridges. The two positions that consistently kept the original residue type of 1kx8 are highlighted in bold. (TIF) [file pcbi.1006623.s005.tif]

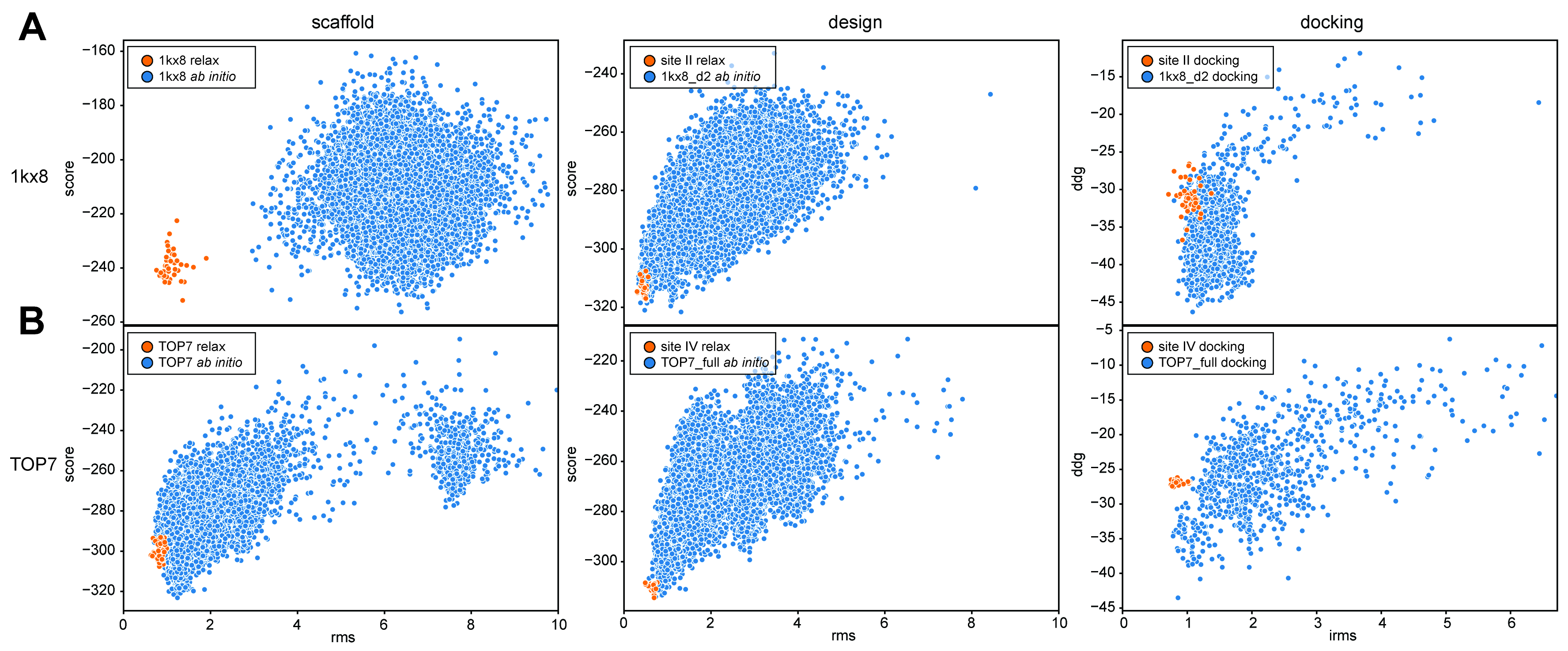

Supplement: S5 Fig — A) Ab initio folding simulations for wild-type 1kx8 (left) and design 1kx8_d2 (center), ensembles generated by relaxing the starting structures are shown in orange. The inability of 1kx8 to form a proper folding funnel could be explained by the big internal cavity of the protein due to its fatty-acid binding pocket. Docking-minimization simulations (right) performed with the top50 scoring ab initio decoys. The docking simulations reveal a similar binding configuration between the peptide motif and the full design, and ΔΔGs are within a similar range to those of the native peptide antibody complex. B) Same simulations as described in A) for wild-type TOP7 (left) and TOP7_full (center). We observe energetically favorable folding funnels for both wildtype and design. The docking simulations showed that the complex between the design and the antibody is formed in a similar structural configuration to the peptide-antibody complex achieving a similar range of ΔΔGs. (TIF) [file pcbi.1006623.s006.tif]

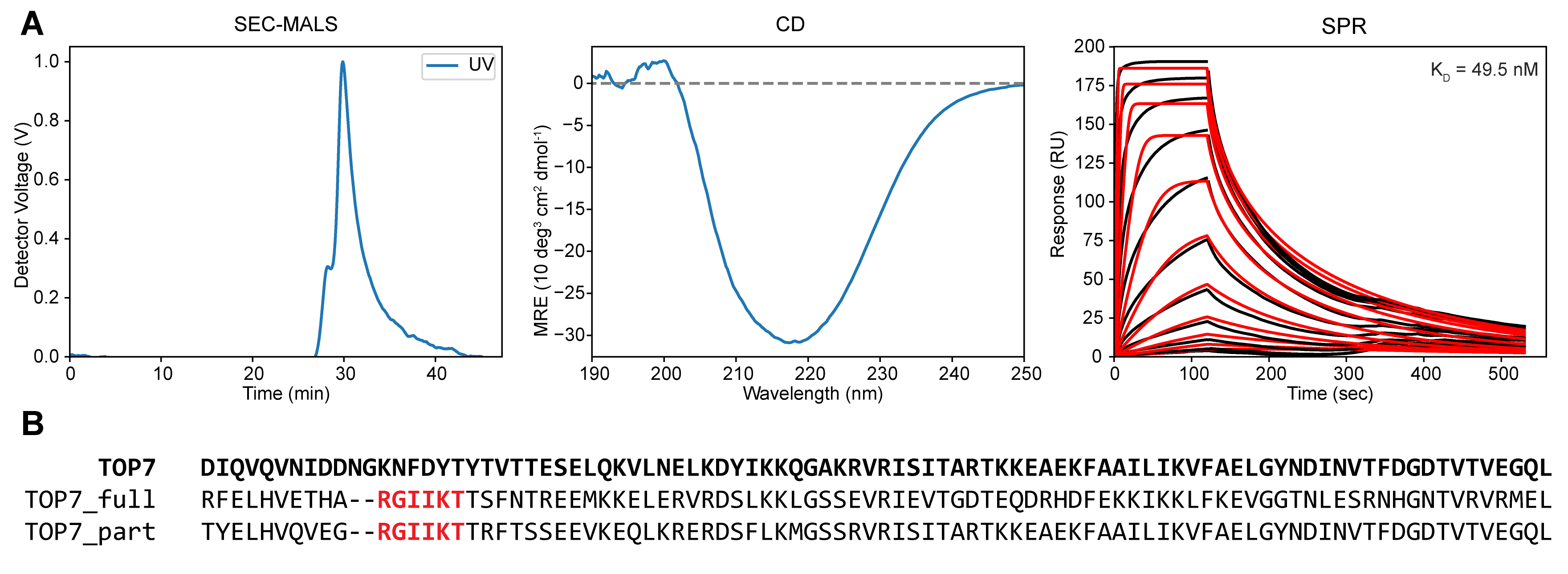

Supplement: S6 Fig — A) Experimental characterization for the TOP7_partial design: SEC-MALS elution profile (left column); CD wavelength scan spectrum; SPR binding assays with the 101F antibody (right column). The TOP7_partial CD spectrum is notably different from WT TOP7 and the TOP7_full design. B) Global sequence alignment of the wild-type protein TOP7 and the computationally designed sequences. Red positions highlight the site IV epitope insertion. (TIF) [file pcbi.1006623.s007.tif]
